# Will climate change increase hybridization risk between potential plant invaders and their congeners in Europe?

**DOI:** 10.1111/ddi.12578

**Published:** 2017-05-31

**Authors:** Günther Klonner, Iwona Dullinger, Johannes Wessely, Oliver Bossdorf, Marta Carboni, Wayne Dawson, Franz Essl, Andreas Gattringer, Emily Haeuser, Mark van Kleunen, Holger Kreft, Dietmar Moser, Jan Pergl, Petr Pyšek, Wilfried Thuiller, Patrick Weigelt, Marten Winter, Stefan Dullinger

**Affiliations:** ^1^ Department of Botany and Biodiversity Research Faculty of Life Sciences University of Vienna Vienna Austria; ^2^ Institute of Social Ecology Faculty for Interdisciplinary Studies Alps Adria University Vienna Austria; ^3^ Institute of Evolution & Ecology University of Tübingen Tübingen Germany; ^4^ Laboratoire d'Écologie Alpine (LECA), CNRS University of Grenoble Alpes Grenoble France; ^5^ Department of Biology, Ecology University of Konstanz Konstanz Germany; ^6^ Department of Biosciences Durham University Durham UK; ^7^ Zhejiang Provincial Key Laboratory of Plant Evolutionary Ecology and Conservation Taizhou University Taizhou China; ^8^ Biodiversity, Macroecology & Biogeography University of Goettingen Göttingen Germany; ^9^ Department of Invasion Ecology Institute of Botany The Czech Academy of Sciences Průhonice Czech Republic; ^10^ Department of Ecology Faculty of Science Charles University Prague Czech Republic; ^11^ German Centre for Integrative Biodiversity Research (iDiv) Halle‐Jena‐Leipzig Leipzig Germany

**Keywords:** alien ornamental plants, climate change, interspecific hybridization, invasion biology, range overlap, species distribution models

## Abstract

**Aim:**

Interspecific hybridization can promote invasiveness of alien species. In many regions of the world, public and domestic gardens contain a huge pool of non‐native plants. Climate change may relax constraints on their naturalization and hence facilitate hybridization with related species in the resident flora. Here, we evaluate this possible increase in hybridization risk by predicting changes in the overlap of climatically suitable ranges between a set of garden plants and their congeners in the resident flora.

**Location:**

Europe.

**Methods:**

From the pool of alien garden plants, we selected those which (1) are not naturalized in Europe, but established outside their native range elsewhere in the world; (2) belong to a genus where interspecific hybridization has been previously reported; and (3) have congeners in the native and naturalized flora of Europe. For the resulting set of 34 alien ornamentals as well as for 173 of their European congeners, we fitted species distribution models and projected suitable ranges under the current climate and three future climate scenarios. Changes in range overlap between garden plants and congeners were then assessed by means of the true skill statistic.

**Results:**

Projections suggest that under a warming climate, suitable ranges of garden plants will increase, on average, while those of their congeners will remain constant or shrink, at least under the more severe climate scenarios. The mean overlap in ranges among congeners of the two groups will decrease. Variation among genera is pronounced; however, and for some congeners, range overlap is predicted to increase significantly.

**Main conclusions:**

Averaged across all modelled species, our results do not indicate that hybrids between potential future invaders and resident species will emerge more frequently in Europe when climate warms. These average trends do not preclude, however, that hybridization risk may considerably increase in particular genera.

## 1 INTRODUCTION

Biological invasions are an important component of global environmental change and may have severe ecological as well as economic impacts (Bellard, Cassey, & Blackburn, 2016; Vilà et al., 2011). Owing to intensified trade and traffic, the global redistribution of species and their subsequent establishment outside their native range (=their naturalization) have considerably increased during the recent decades and are likely to further increase in the future (Seebens et al., 2015). Pro‐active management of such invasions is, however, hampered by the difficulty of predicting which species may become invasive and where. Such predictions are difficult because of the complex causes of invasions, which include biological traits of the invading species, biotic and abiotic characteristics of the recipient environment, and historical contingencies (Catford, Jansson, & Nilsson, 2009; Richardson & Pyšek, 2006). There are, however, a number of factors known to facilitate invasions such as early reproduction, rapid growth rate, efficient long‐distance dispersal or specific trait profiles which are complementary to those of the resident biota (Buhk & Thielsch, 2015; Carboni et al., 2016; van Kleunen, Weber, & Fischer, 2010; Küster, Kühn, Bruelheide, & Klotz, 2008; Pyšek et al., 2015).

Apart from these factors, interspecific hybridization has been assumed to foster invasions since a seminal paper of Ellstrand and Schierenbeck (2000). Indeed, there are prominent examples of highly invasive hybrids. For instance, several species of the genus *Tamarix* have been introduced to North America during the 19th century. Although all of these species have escaped cultivation, by far the most successful and widespread invader is the hybrid between *T. ramosissima* × *T. chinensis* (Gaskin & Kazmer, 2009; Gaskin & Schaal, 2002). The same Eurasian *T. ramosissima* has recently started to hybridize with native *T. usneoides* in South Africa (Mayonde, Cron, Gaskin, & Byrne, 2015). Other examples of genera that have produced successful invasive hybrids include *Rhododendron* (Milne & Abbott, 2000), *Spartina* (Thompson, 1991), *Senecio* (Abbott et al., 2009) and *Helianthemum* (Rieseberg et al., 2007). More generally, the idea that interspecific hybrids may be especially successful invaders has been corroborated by a recent meta‐analysis (Hovick & Whitney, 2014). The possible reasons for hybrid success include increased phenotypic or genotypic variability, phenotypic novelty arising from transgressive segregation or adaptive introgression, and heterosis effects (Prentis, Wilson, Dormontt, Richardson, & Lowe, 2008). Heterosis effects may be maintained especially when hybridization is accompanied by allopolyploidization and/or a shift to apomictic reproduction, which sustain heterozygosity.

As species are transported around the world with increasing intensity, barriers to gene flow between once geographically separated species are reduced and new hybrids between introduced and resident species will probably emerge more frequently (Thomas, 2013). For the British Isles, a recent overview has already demonstrated a rise in the number of hybrids during the last few decades (Stace, Preston, & Pearman, 2015). Apart from the risk that the new hybrids include particularly successful future invaders, rising hybridization rates also raise conservation concerns (Bohling, 2016). In particular, genetic introgression and outbreeding depression may severely threaten native species (Todesco et al., 2016), especially those that are rare and only exist in small populations (Bleeker, Schmitz, & Ristow, 2007).

Disregarding deliberate crossings (e.g., for horticultural reasons), the risk of hybridization between introduced and resident species will depend on the introduced species' ability to naturalize, that is to establish self‐sustaining populations in the wild, because naturalization intensifies the spatial contact of the newcomers with their potential hybridization partners in the regional flora and hence increases mating opportunities. The likelihood of naturalization of an introduced species is mainly determined by propagule pressure (Simberloff, 2009) and the suitability of abiotic and biotic conditions (Pyšek et al., 2012; Shea & Chesson, 2002). Among the abiotic factors, climatic suitability has been repeatedly shown to play a prominent role (e.g., Feng et al., 2016; Hayes & Barry, 2007; Thuiller et al., 2005). As a corollary, predicted climate change is also likely to alter the naturalization odds of introduced alien species and thus the likelihood that they hybridize with resident species (e.g., Bellard et al., 2013).

The alien flora of a region consists, first, of plant species that have already become naturalized or invasive. In addition, there is an often much larger group of alien species that have been introduced to a region and are grown there but have not escaped from cultivation yet. The latter group of species forms a massive pool of potential future additions to the regional wild flora. In many regions, this pool is dominated by non‐native plants used for public and domestic gardening (Hulme et al., 2008; Niinemets & Penuelas, 2008; Pergl et al., 2016). In Europe, for example, more than 16,000 species from more than 200 families are currently in cultivation for ornamental purposes, with many of them being alien to Europe (Cullen, Knees, & Cubey, 2011). Some of these non‐native garden plant species have already become naturalized or invasive elsewhere in the world (van Kleunen et al., 2015) and can hence be considered particularly likely to do so in Europe too (Williamson, 1999).

In a recent paper, Dullinger et al. (2016) showed that this latter group of “alien garden plants naturalized elsewhere” will benefit from a changing climate in Europe in as much as the area climatically suitable to them will increase. Given that climatic suitability is an important prerequisite to alien species' naturalization and that naturalization facilitates hybridization of introduced and resident species, the risk that new hybrids emerge may thus also be expected to increase in the future. The newly establishing garden plants may thereby hybridize with resident (i.e., native and already naturalized or even invasive) species (e.g., Ayres, Smith, Zaremba, Klohr, & Strong, 2004). However, a climate‐driven modification of regional hybridization risk does not only depend on the naturalization odds of garden plants, but also on changes in climatically suitable ranges of their potential hybridization partners (Dehnen‐Schmutz, 2011). In other words, the changing spatial overlap in areas climatically suitable for alien garden plants and for their potential resident hybridization partners in the wild (both native and naturalized) flora will determine possible changes in the risk of hybridization between these two groups.

Here, we evaluated whether climate change may lead to an increase in this spatial overlap. We studied a group of 783 alien ornamental plants not yet naturalized in Europe, but established outside their native range elsewhere in the world, as identified in Dullinger et al. (2016). From this group of 783 species, we first selected all those belonging to genera with hybridization documented in the literature. We then fitted species distribution models for this subset of non‐native ornamentals as well as for all their congeners in the native and naturalized European flora. We restricted our analysis to congeners because hybridization risk is strongly linked to genetic distance (Mallet, 2005), and intergeneric hybrids are rare (Whitney, Ahern, Campbell, Albert, & King, 2010). Finally, we assessed to what extent the range matching between the selected garden plants and their congeners will increase under three different climate change scenarios.

## 2 METHODS

### 2.1 Species selection

Our initial pool of study species was the same as used by Dullinger et al. (2016). These authors aligned the European Garden Flora (EGF; Cullen et al., 2011), the most comprehensive encyclopaedia of ornamental plants in Europe, with the Global Naturalised Alien Flora (GloNAF; van Kleunen et al., 2015; https://glonaf.org/), a global database of naturalized alien plant species. They thereby identified non‐native ornamental plants cultivated in Europe which have naturalized somewhere outside of Europe, but not yet in Europe. For species distribution modelling (SDM) purposes, this list was then reduced to those 783 species with more than 50 occurrences found in a search of the Global Biodiversity Information Facility (GBIF, http://www.gbif.org/) database.

Here, we used a systematic web‐based literature search to further narrow this group of candidate species to those particularly relevant in the context of both invasion and hybridization. We used all possible combinations of the following keywords in the Web‐of‐Science (http://apps.webofknowledge.com): #hybridization, #hybridisation, #invasion, #alien, #invasive species, #plant. The records were subsequently limited to the following categories: agriculture, biodiversity, conservation, ecology, environmental sciences, evolutionary biology and reproductive biology. We screened the abstracts of the 1,220 papers found and finally identified 66 plant genera that fulfil the following criteria: (1) interspecific hybridization has been documented and (2) they contain invasive species (even if these are not identical with the hybrids or if only intraspecific hybrids have so far been reported to be invasive, for example in *Pyrus* (Hardiman & Culley, 2010)). Twenty‐three of these genera were represented by at least one species in the list of Dullinger et al. (2016), of which 18 were also represented by at least one species (native and naturalized) in the flora of Europe (Tutin et al., 1964–1980). From these, we discarded the genera *Rosa* and *Rubus* because of taxonomic difficulties with a large number of apomictic species. As a result of these consecutive filtering steps, we ended up with 16 genera. These 16 genera contain 34 alien plants currently cultivated in Europe with the potential to escape into the wild (indicated by their naturalization in other continents) and at least one congeneric species in the native and naturalized flora of Europe which shares the same life form (assuming that only mating partners of the same life form are likely to produce viable hybrid offspring; see Tables S1, S3, S6). Most of these species are planted for ornamental purposes only, but some, like *Chenopodium quinoa* or several *Eucalyptus* spp., are also of commercial interest beyond horticulture. After a final screening in GBIF for those species with more than 50 occurrence records (see Table S2), the group of congeneric species within Europe contained 133 native and 40 alien naturalized spp (see Table S6).

### 2.2 Species distribution data and climatic maps

Data on the world‐wide distribution of the 34 alien garden plants and their 173 native and naturalized congeners were taken from GBIF. All species lists were taxonomically harmonized using The Plant List (http://www.theplantlist.org). Multiple occurrences within 10′ × 10′ grid cells and clearly erroneous records, that is those in water bodies, were removed. We did not limit records to those from the native range because species are known to partly expand their realized climatic niches in the naturalization range (Dellinger et al., 2016; Early & Sax, 2014; Petitpierre et al., 2012).

For characterizing the means and annual variability of the current temperature and precipitation patterns, we used six bioclimatic variables (climatic data averaged for the baseline period 1950–2000) provided by WorldClim (Hijmans, Cameron, Parra, Jones, & Jarvis, 2005): BIO4—Temperature Seasonality, BIO5—Max Temperature of Warmest Month, BIO6—Min Temperature of Coldest Month, BIO16—Precipitation of Wettest Quarter, BIO17—Precipitation of Driest Quarter, BIO18—Precipitation of Warmest Quarter. All these variables are known to potentially influence species distributions (Root et al., 2003). All climatic variables were provided by worldclim at a spatial resolution of 10 min.

Possible future climates in Europe were represented by three emission scenarios of the IPCC5‐scenario family: the milder RCP2.6, the medium RCP4.5 and the severe RCP8.5 (IPCC, 2013). The respective monthly temperature and precipitation time series, already regionalized for Europe, were taken from the Cordex portal (http://cordexesg.dmi.dk/esgf-web-fe/live) and used to recalculate 10′ resolution maps of the above six bioclimatic variables for possible future climates of the 21st century. A 50‐year average of the period 2050–2100 was then used as the climate of the future in model projections (see below).

### 2.3 Species distribution models

We used the biomod2 platform (Thuiller, Lafourcade, Engler, & Araujo, 2009) in R (R Core Team, 2015) to quantify species' climatic niches and subsequently project current and future spatial distributions. The following modelling algorithms were used: generalized linear model (GLM), general additive model (GAM), boosted regression tree (BRT) and random forest (RF). For applying these species distribution models (SDMs) with presence‐only data as provided by GBIF, we generated “pseudo‐absences” following recommendations of Barbet‐Massin, Jiguet, Albert, and Thuiller (2012): for regression technique models (GLM and GAM), we used 10,000 randomly distributed absences, and for machine‐learning technique models (BRT and RF), we used a number of pseudo‐absences equal to the number of occurrences found in GBIF and selected outside a radius of 200 km around these occurrences. In the latter case, pseudo‐absence generation, and hence model calibration, was repeated 10 times per species to ensure that selected pseudo‐absences did not bias the final predictions. For all models, the weighted sum of presences equalled the weighted sum of pseudo‐absences. The predictive performance of the models was evaluated by means of the true skill statistic (TSS; Allouche, Tsoar, & Kadmon, 2006) based on a repeated (three times) split‐sampling approach in which models were calibrated with 80% of the data and evaluated over the remaining 20%. Evaluated models were then used for two different average projections of the spatial distribution of each of the 34 garden plants and their 173 native and naturalized congeners under current climatic conditions and the three climate change scenarios: one comprised the two regression‐based techniques and one comprised the two machine‐learning techniques. The probabilistic output of the two ensemble models was aggregated to a weighted mean, with weights determined by their respective TSS scores. Similarly, binary outputs of each of the two ensemble projections were generated based on a threshold that maximizes the TSS score (Liu, Berry, Dawson, & Pearson, 2005; Liu, White, & Newell, 2013) and then aggregated to a conservative consensus ma; that is, 10′ resolution cells were only classed as climatically suitable to a species if both ensemble models agreed on the potential presence of the species in the cell.

### 2.4 Overlap of climatically suitable ranges

Geographic overlap between the climatically suitable ranges of the 34 alien garden plants and their 173 congeners under current and future climatic conditions was quantified by calculating the TSS from binary projections. Further, range overlap was quantified by the total number of overlapping grid cells, again based on binary projections. Both metrics were calculated for each possible species pair; that is, each of the 34 garden plants was combined with any of its congeners. Overlap metrics were subsequently averaged per species of garden plant (i.e., the average range overlap of each garden plant species and all its congeners in the wild flora was computed), separately for each climate change scenario. These average overlaps were then compared among the current climate and each climate change scenario using linear mixed‐effects models (LMMs). Each LMM used the 34 ratios of current‐to‐future climatic range overlaps as the response, which was regressed against a fixed intercept, that is we tested whether the mean of the logarithm of these ratios was significantly larger or smaller than 0. A random intercept for genus was estimated to account for the fact that some genera were represented by more than one species of garden plant.

All analyses were carried out in R (R Core Team, 2015) mainly using the packages raster (Hijmans & van Etten, 2012) for handling of SDM gridded outputs, presenceabsence (Freeman & Moisen, 2008) for calculating TSS and evaluation metrics and nlme (Pinheiro, Bates, DebRoy, Sarkar, & Team, 2015) for LMMs.

## 3 RESULTS

### 3.1 Geographic overlap of suitable ranges

Species distribution models for both the 34 alien garden plants and their 173 congeners in the native and naturalized European flora produced accurate projections in most cases (see Table S6).

**Figure 1:**
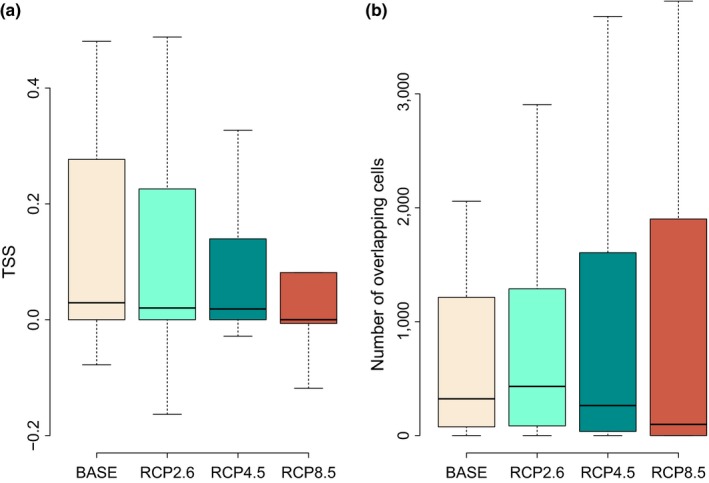
Mean overlap in areas climatically suitable to 34 alien garden plants and their congeners in the native and naturalized flora of Europe. Overlap was quantified by the true skill statistic‐TSS (a), or the number of overlapping cells (b), and calculated for current climate (BASE) and under three scenarios of climate change (RCP2.6, RCP4.5, RCP8.5) for the second half of the 21st century (2050–2100)

True skill statistic scores suggest that the mean geographical overlap between the climatically suitable ranges of the 34 garden plants and their congeners will decrease under a warming climate (Figure 1a): the overlap is lowest under the strongest scenario (RCP8.5) and also significantly different from current climatic conditions under the mild and intermediate scenarios (RCP2.6 and RCP4.5; see Table S4). When overlap is measured as the number of 10′ × 10′ cells that are climatically suitable to both the garden plants and their congeners (i.e., the absolute size of their overlapping range, see Table S5), the results suggest that a warmer climate will not change the size of overlapping ranges in a statistically significant way in any of the scenarios (Figure 1b, see Table S4).

**Figure 2:**
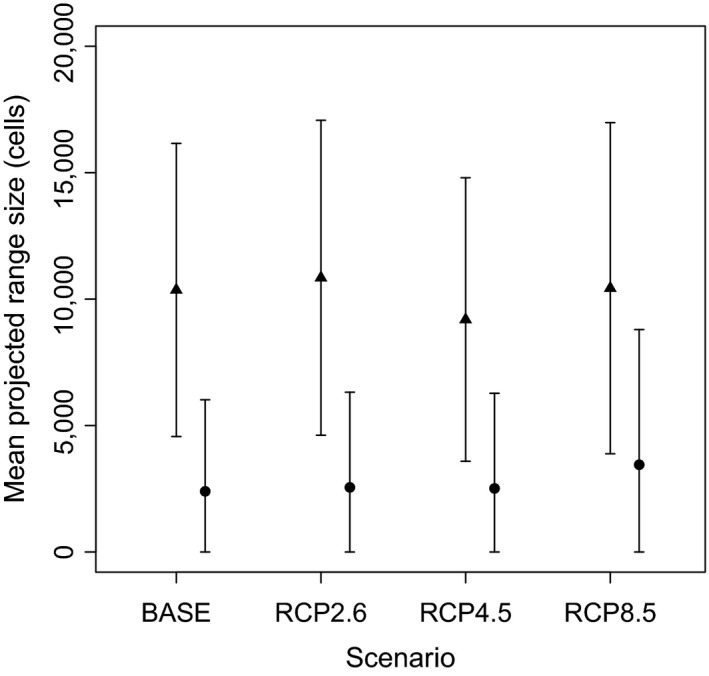
Mean projected range size of 34 alien garden plants (circles) and of their 173 congeners in the native and naturalized flora of Europe (triangles) under current climate (BASE) and under three different scenarios of climate change (RCP2.6, RCP4.5, RCP8.5) for the second half of the 21st century (2050–2100). The error bars indicate the standard deviation

Looking at climatically suitable ranges of the 34 garden plant species and their 173 congeners separately indicates that these results are partly driven by opposite effects of climate change on the two species groups: while average range size (=number of suitable cells) is projected to increase for the garden plants (statistically significantly only for scenario RCP8.5, see Table S4), it will remain constant or even decrease for their congeners in the wild European flora, at least under the more severe scenarios (RCP4.5 and RCP8.5, Figure 2 and see Table S4). These opposite trends apparently result in no net change in overlap or in a slight reduction depending on scenario and overlap measure used, but never in a significant increase in overlap.

**Figure 3:**
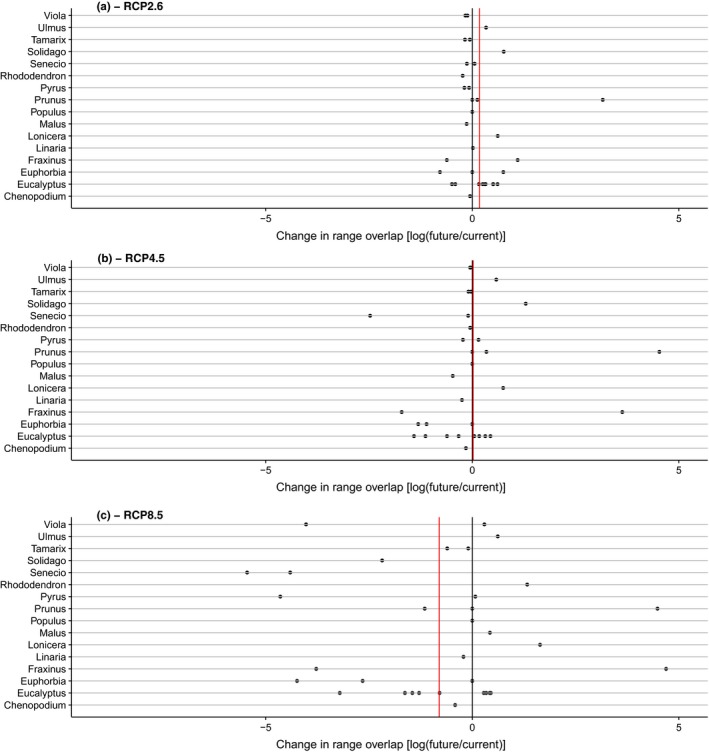
Change in overlap of areas climatically suitable to 34 alien garden plants and their 173 congeners in the native and naturalized flora of Europe. Overlap in areas is measured by the log of the ratio of the number of 10 × 10′ cells suitable to both species in a possible species pair. Each point represents the average change in overlap between one of the 34 garden plants and all its congeners under the respective climate scenario (some points represent more than one pair because of identical values). Values <0 represent a decrease, values >0 an increase, values = 0 no change in overlap. The three panels refer to climate change scenarios RCP2.6 (a), RCP4.5 (b) and RCP8.5 (c). The red line represents the mean over all pairs

These average trends mask strong differences among genera. Figure 3 demonstrates that the number of cells climatically suitable to both the 34 garden plants and their European congeners can either strongly decrease or increase under each of the future climate scenarios, and variation among individual species pairs (i.e., a particular garden plant species with all its individual congener species) is even more pronounced. In particular, under each of the scenarios, there are a number of genera for which spatial overlap of suitable ranges between non‐native ornamental plants and their European congeners will increase markedly. This is especially true for the genera *Solidago*,* Fraxinus*,* Lonicera* and *Prunus*.

## 4 DISCUSSION

Taken together, our results do not support the expectation that the area suitable to both the group of potential future invaders among European garden plants and their congeners in the resident flora of the continent will increase under a changing climate. Potential range overlap between these two groups of species will rather decrease under all warming scenarios. This is partly due to opposing trends in the size of climatically suitable ranges among the two groups: while potential invaders on average expand their suitable ranges, those of resident congeners remain constant or shrink, at least under moderate and severe warming. However, there is pronounced variation among the different species pairs and for some of them the predicted increase in range overlap is significant, suggesting that the risk of hybridization between them will also increase.

Climate change has already allowed many alien species to expand their non‐native ranges (e.g., IPCC, 2014). For ornamental plants, the main reason for this trend is probably their widespread commercial use beyond climatic conditions they would tolerate in the wild, which gives them a head start when the climate warms (Van der Veken, Hermy, Vellend, Knapen, & Verheyen, 2008). Predictions of increasing suitable range sizes of ornamental plants in a warming Europe likely have similar underlying reasons. Many ornamentals currently cultivated on the continent come from warm(er) regions and hence tend to expand towards north‐eastern and north‐western Europe, in particular, if climatic constraints in these regions are relaxed (cf. Bellard et al., 2013; Dullinger et al., 2016). The 34 non‐native ornamental plants used in this study are also mostly native to warm regions and hence their potentially suitable ranges in Europe tend to increase, on average, despite pronounced idiosyncratic differences. Although the pool of their European congeners contains many warm‐adapted species too (e.g., most species from the genera *Euphorbia* and *Tamarix*), it also includes a considerable number of montane or even alpine species (e.g., from the genera *Linaria*,* Rhododendron*,* Senecio* and *Viola*). For montane species, climatically suitable ranges are particularly likely to shrink under climate warming (Engler et al., 2011; Thuiller et al., 2014). The share of montane species is thus probably a factor restricting range increases of congeners in the more severe climate scenarios.

We emphasize that our estimate of changing range overlaps does not include a temporal dimension. Real changes in overlap of species distribution over the 21st century may actually deviate from those projected here. On the one hand, wild populations of species (both native and naturalized) will likely lag behind the changing climate due to dispersal and migration constraints (e.g., Corlett & Westcott, 2013; Dullinger et al., 2015). These constraints are less relevant or even irrelevant for ornamental plants in horticultural trade. Actually, garden plants may even “overtake” climate change when regional demand of gardeners anticipates future climatic alterations (Bradley et al., 2012). On the other hand, remnant populations of species in the wild may still occupy an area long after the average climate has become unsuitable to them (Eriksson, 2000). Actual range overlap over the next decades will hence not only be a function of changes in suitable ranges, but will be co‐determined by the behaviour of gardeners and by migration lags and extinction debts of wild populations (Dullinger et al., 2012). Thus, we may expect that our SDM‐based projections will underestimate real overlap near the wild species' trailing edges (because of delayed extinctions), but overestimate it near the wild species' leading edges (because of lagged migration).

An average decrease in range overlap among all the species pairs tested here does not necessarily imply a general decrease in hybridization risk from invasive plants in Europe. First, we deliberately restricted our approach to hybridization among potential future invaders and resident species but did not consider the possible emergence of hybrids within the resident (i.e., native and already naturalized or even invasive) species. Among the latter, several hybrids come from genera well‐known to hybridize such as *Fallopia* (Parepa, Fischer, Krebs, & Bossdorf, 2014) or *Epilobium* (Gregor et al., 2013). For an exhaustive evaluation of climate‐driven changes in hybridization risk of non‐native plants, these species would have to be included into the models. Second, the probability of hybridization risk will likely vary widely among the species pairs included in this study. Successful establishment of allopolyploid hybrids, for example, depends on plant traits (Mallet, 2007). In addition, the genetic distance between species certainly differs a lot among the pairs studied and hence also the likelihood that reproductive barriers break down (Mallet, 2005). A more precise evaluation of hybridization risk under climate warming would therefore have to weight changing range overlaps by the likelihood that particular species pairs hybridize at all—and, in an additional step, by the probability that a particularly successful invader emerges from such hybridization (e.g., Abbott et al., 2009; Hovick & Whitney, 2014). Such weighting might significantly modify expected changes in hybridization as individual species pairs with increasing range overlap are to be found in almost all genera. Although data for reliable estimation of these weights are lacking, we emphasize that among the genera with increasing average range overlaps in at least some scenarios, species in *Solidago* and *Rhododendron* have already produced invasive hybrids in Europe (Abbott et al., 2009; Erfmeier, Tsaliki, Ross, & Bruelheide, 2011; Karpaviciene & Radusiene, 2016) and may hence be particularly likely to do so in the future again. In addition, among the genera which were both identified to have produced invasive hybrids in the meta‐analysis of Hovick and Whitney (2014) and used in our study, three include species pairs with increasing average range overlaps in at least some climate change scenarios (*Rhododendron*,* Ulmus*,* Viola*) and only one solely contains pairs with decreasing average overlap (*Tamarix*).

Although we consider the change in suitable range overlap to be a sensible indicator of changing hybridization risk, the emergence of hybrids does not necessarily depend on the contact of the species in the wild. Some of the native or already naturalized congeners in our study are species that frequently occur at ruderal sites or even as garden weeds (e.g., *Euphorbia peplus*,* Senecio vulgaris*) and hence also potentially reproduce with plants cultivated in gardens or parks. For these species, changing hybridization risk might more realistically be estimated from how their future suitable ranges overlap with the possible area where potential hybridization partners among ornamental plants can be cultivated when climate warms. These areas are usually much larger than those suitable for establishment of wild populations (Van der Veken et al., 2008) and hence risk assessments based on the latter may actually be underestimates.

Apart from potentially fostering invasiveness, hybridization between alien and native plants may threaten native populations of rare species through outbreeding depression (Bleeker et al., 2007), gene swamping (Todesco et al., 2016) or pollen competition (Arceo‐Gomez & Ashman, 2016). Among the genera included in this study, introgressive hybridization has been documented in several cases (e.g., *Tamarix* (Gaskin & Kazmer, 2009), *Rhododendron* (Stace et al., 2015), *Viola* (Stace et al., 2015)). Conversely, Bleeker et al. (2007) have identified 18 native species red‐listed in Germany, which potentially suffer from outbreeding depression when hybridizing with more abundant aliens. Among the 13 genera these species belong to, six are also included in our study (*Euphorbia*,* Malus*,* Populus*,* Prunus*,* Solidago*,* Viola*) with two of them (*Solidago*,* Prunus*) tending towards increased range overlap with native congeners under a warming climate (these results are very similar when the climatic area of natives that is also suitable to their non‐native congeners among garden plants is calculated as a measure of threat to the native plants, see Fig. S3). In addition, Bleeker et al. (2007) listed threatened native *Viola* spp. as sensitive to gene introgression from alien congeners. Similar evaluations for other European countries are largely lacking. However, across Europe, the congeners of our 34 potential future invaders include many regionally endangered or even globally rare species such as Mediterranean endemics in the genera *Linaria*,* Senecio* or *Viola*. Although the magnitude of threat to rare species from outbreeding depression and introgression with hybridizing aliens is not well documented yet (Bohling, 2016), future escape and expansion of ornamental plants into the range of these endemics may actually put additional pressure on them, beyond the challenges they face under a warming climate. Most of these species are not included in our study as their distribution is not represented well enough in GBIF, but this issue certainly warrants further investigation.

Finally, as a last caveat, we note that the models this study is based on were fitted using data taken from GBIF. This source combines the advantage of a global coverage, and hence the possibility to fit niches of species comprehensively, with the disadvantage of the errors and biases implicit to this database (Meyer, Weigelt, & Kreft, 2016). Uncertainties in species distribution estimates and models resulting from these caveats have a clear geographical bias and are least pronounced in the well represented regions of Europe, North‐ and Central America, and Australia (Meyer et al., 2016). The majority of the ornamental plants and all congeners modelled here come from these areas, and we hence assume that data problems are of limited importance for them. Several of the ornamental plants are native to temperate Asia and Africa, however, and these regions have notoriously low data coverage. The most likely consequence of this low coverage is an underestimate of these species' niches and hence of their potential distribution in Europe as well as their overlap with native and already naturalized congeners. Such underestimation may have been reinforced by the restrictive rules of our consensus projections. As a result, range overlap estimates computed here are probably conservative. We do not, however, think that these data problems affect our main result, namely that the average potential range overlap between ornamental plants and congeners does not increase under a warming climate. This is because predicted trends for species of Asian and African origin are similar to those of the remaining species (see Fig. S1 and Fig. S2 respectively).

## 5 CONCLUSION

Climate warming will potentially increase the area suitable for the naturalization of many non‐native ornamental plants in Europe (Dullinger et al., 2016), but the mean geographical overlap of climatic ranges between the selection of ornamentals and their native and naturalized congeners modelled here is unlikely to increase in the future. Thus, the average risk that garden plants and their wild congeners in the European flora will hybridize does not appear to rise when climate warms. We emphasize, however, that suitable range overlaps do increase for many individual congener pairs and that the pair‐specific likelihood of successful hybrid establishment is unknown. A decreasing average range overlap does not, therefore, preclude increasing invasion risk from hybrids between particular species pairs.

## AUTHOR CONTRIBUTIONS

S.D. and G.K. conceived the ideas; G.K. and A.G. analysed the data; I.D. and J.W. contributed to the analyses; G.K. and S.D. led the writing of the manuscript; all authors contributed to the discussion of ideas and revised the text.

## BIOSKETCH

**Günther Klonner** is a Ph.D. student at the Department of Botany and Biodiversity Research, University of Vienna. His research interests are large‐scale patterns in plant invasion especially the role of plant traits and enhanced modelling of climate‐driven distributions.
