# Supplementary figures and images for: Will climate change increase hybridization risk between potential plant invaders and their congeners in Europe?
Source: Divers Distrib. 2017 May 31;23(8):934–43. doi: 10.1111/ddi.12578 (PMC5518762; doi:10.1111/ddi.12578)

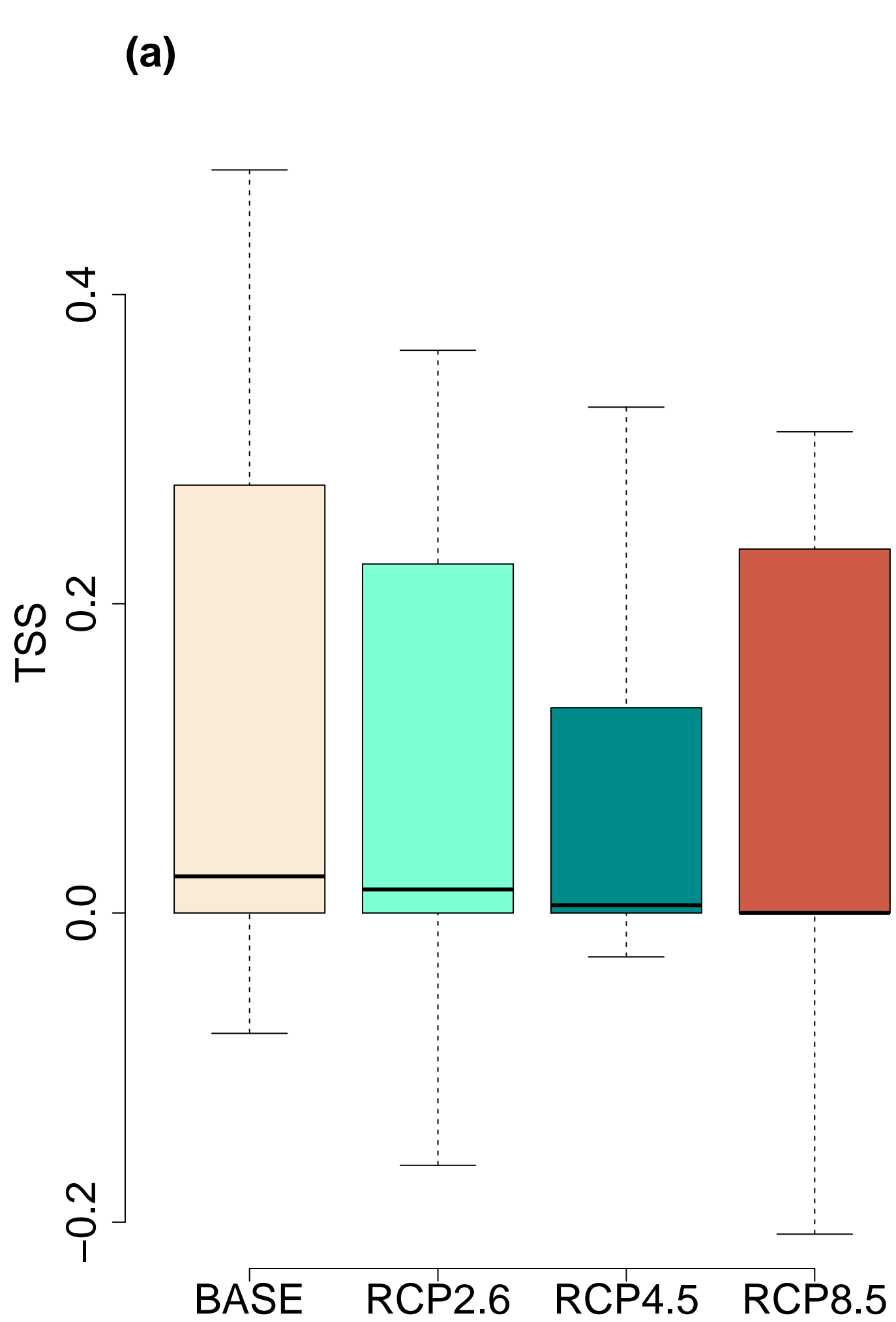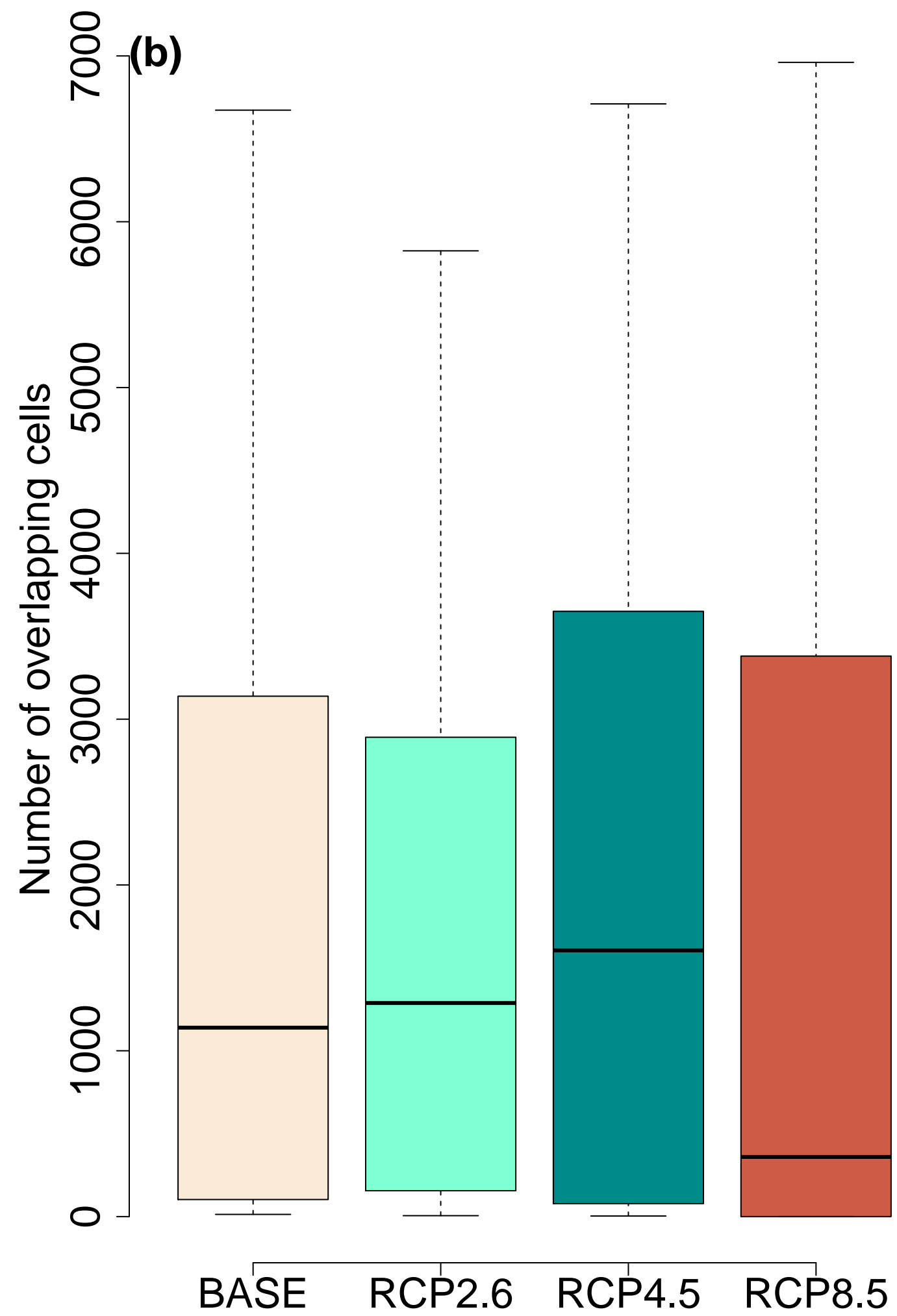

Supplement: Supplementary file 1 [file DDI-23-934-s001.pdf]

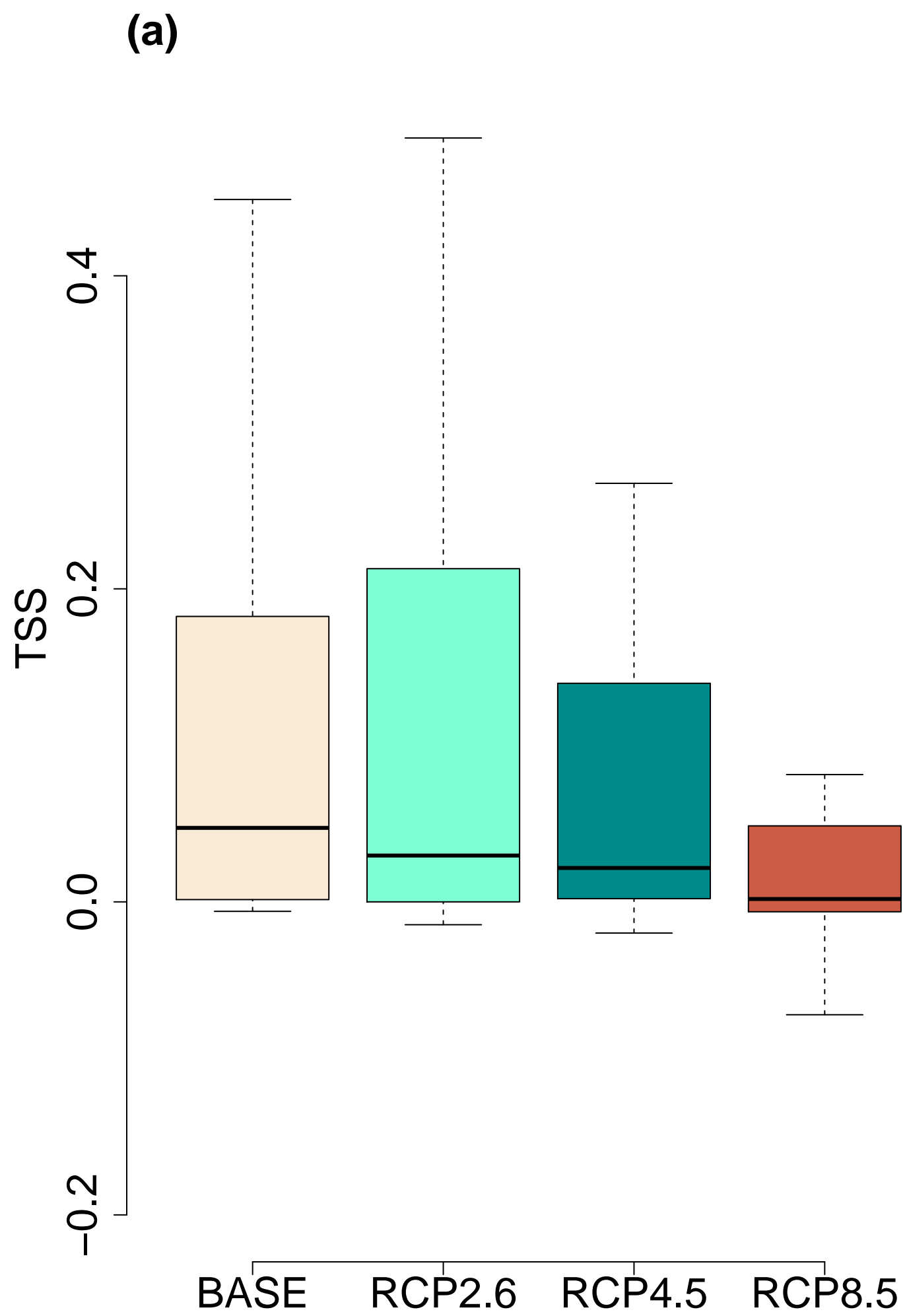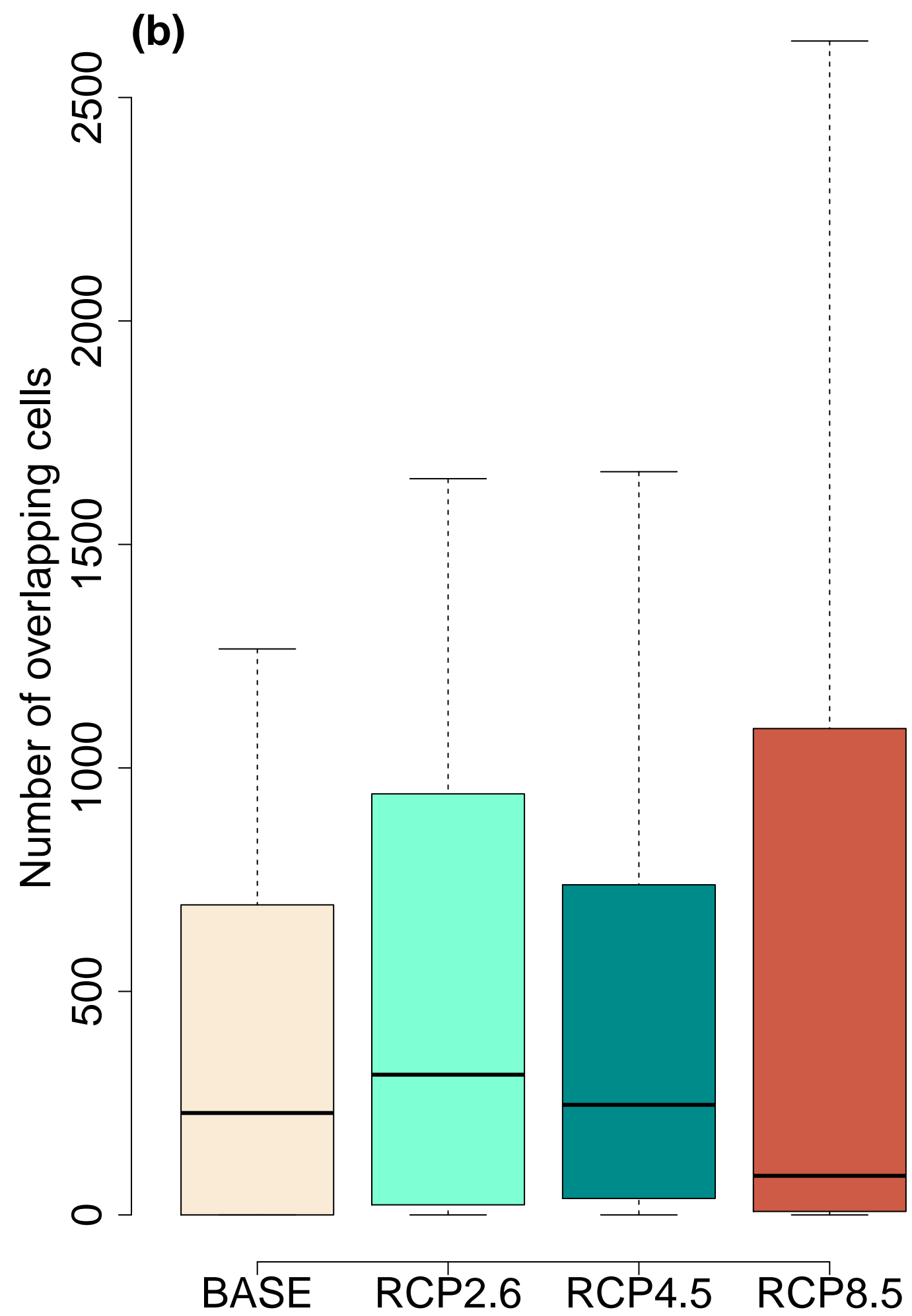

Supplement: Supplementary file 2 [file DDI-23-934-s002.pdf]

**(a) – RCP2.6**

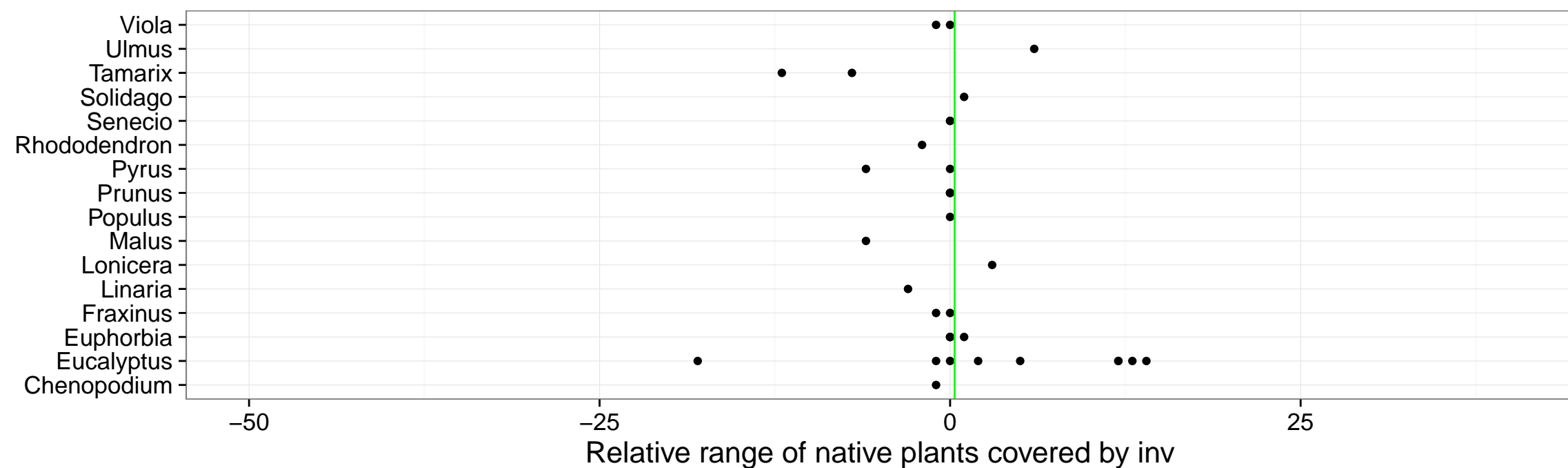

**(b) – RCP4.5**

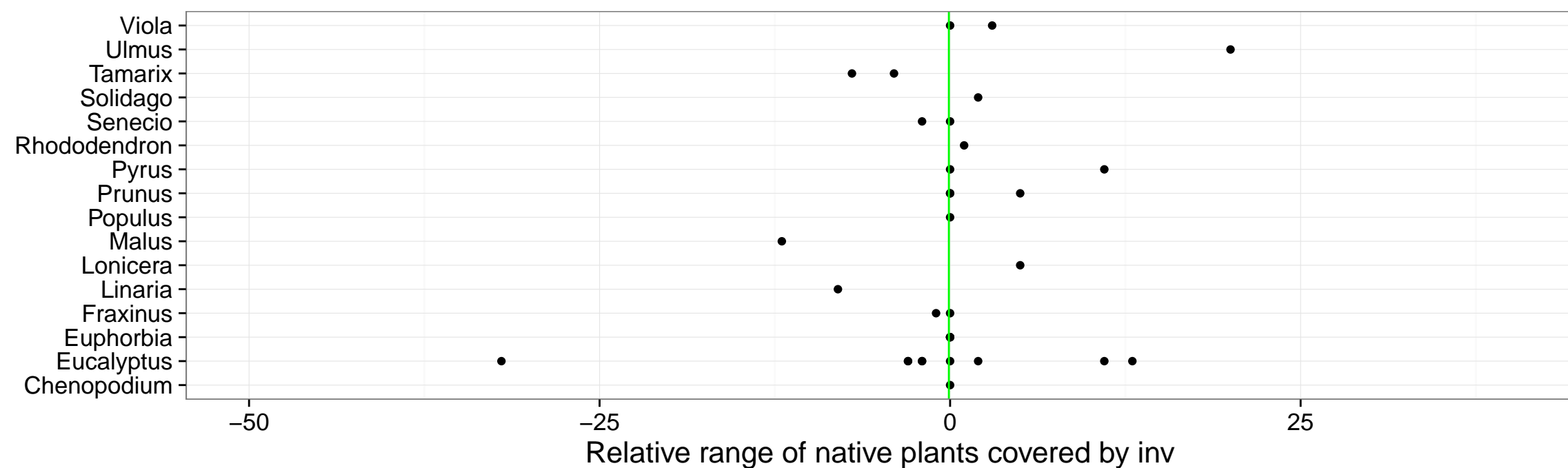

**(c) – RCP8.5**

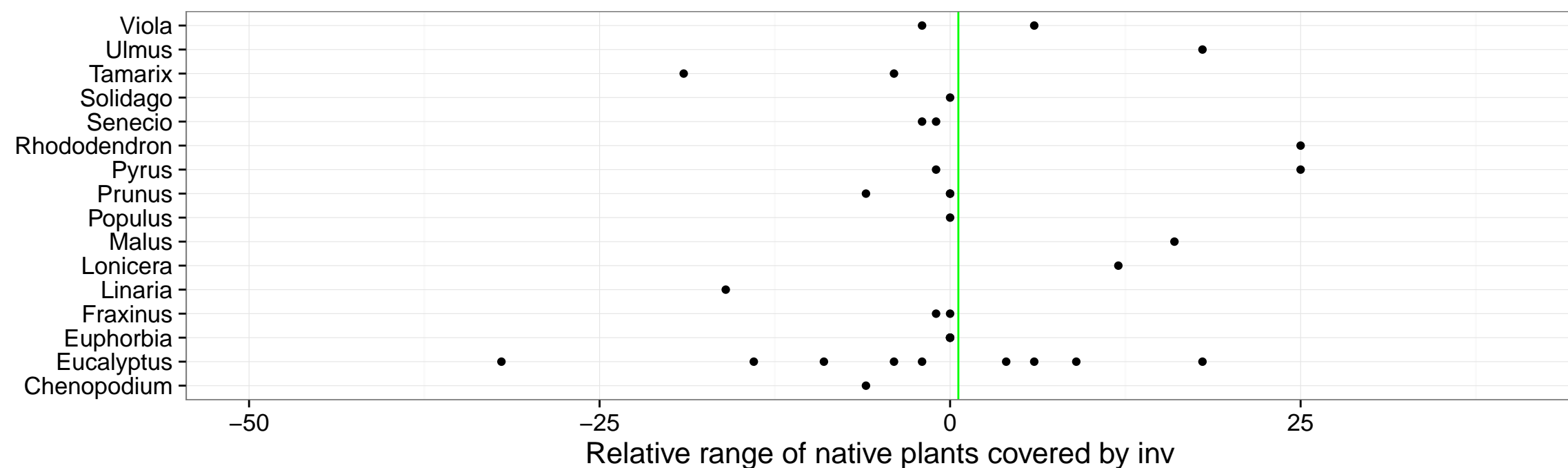

Supplement: Supplementary file 3 [file DDI-23-934-s003.pdf]
