# Supplementary material for: Will climate change increase hybridization risk between potential plant invaders and their congeners in Europe?
Source: Divers Distrib. 2017 May 31;23(8):934–43. doi: 10.1111/ddi.12578 (PMC5518762; doi:10.1111/ddi.12578)
Supplement: Supplementary file 4 [file DDI-23-934-s004.docx]

**(A) APPENDIX**

Table S1: Overview of plant genera for which interspecific hybridization is documented, which contain garden plant species cultivated in Europe and naturalized somewhere in the world but not yet in Europe, which have at least one congener in the native or already naturalized European flora, and which are represented by > 50 occurrence records in GBIF. The taxonomically difficult genera *Rosa* and *Rubus* were excluded. FE indicates the number of congeneric species in the native or already naturalized European flora according to the Flora Europea (Tutin et al. 1964-1980). “Hybrid” indicates whether a genus contains interspecific hybrids that have naturalized somewhere (1). In addition, examples of references to articles in ISI-listed journals that report successful interspecific hybridization are given under “source”.

| **genus** | **FE** | **hybrid** | **source** |
| --- | --- | --- | --- |
| *Chenopodium* | 27 | 1 | e.g. (Bleeker *et al.*, 2007) |
| *Eucalyptus* | 11 | 0 | e.g. (Barbour *et al.*, 2006, 2007; 2010) |
| *Euphorbia* | 106 | 1 | e.g. (Bleeker *et al.*, 2007) |
| *Fraxinus* | 5 | 1 | e.g. (Thomasset *et al.*, 2014) |
| *Linaria* | 70 | 1 | e.g. (Ward *et al.*, 2009) |
| *Lonicera* | 17 | 1 | e.g. (Bleeker *et al.*, 2007) |
| *Malus* | 6 | 1 | e.g. (Bleeker *et al.*, 2007) |
| *Populus* | 11 | 1 | e.g. (Bleeker *et al.*, 2007) |
| *Prunus* | 21 | 1 | e.g. (Bleeker *et al.*, 2007) |
| *Pyrus* | 12 | 0 | e.g. (Yamamoto *et al.*, 2002; Hardiman & Culley, 2010; Bell & Itai, 2011) |
| *Rhododendron* | 6 | 1 | e.g. (Erfmeier *et al.*, 2011) |
| *Senecio* | 67 | 1 | e.g. (Pelser *et al.*, 2012) |
| *Solidago* | 5 | 1 | e.g. (Bleeker *et al.*, 2007) |
| *Tamarix* | 14 | 1 | e.g. (Gaskin & Schaal, 2002; Gaskin & Kazmer, 2009; Lindgren *et al.*, 2010; Mayonde *et al.*, 2015) |
| *Ulmus* | 6 | 1 | e.g. (Zalapa *et al.*, 2010) |
| *Viola* | 92 | 1 | e.g. (Bleeker *et al.*, 2007) |

Table S2: List of model species (i.e. those alien garden plants cultivated in Europe and naturalized somewhere in the world but not yet in Europe, which have at least one congener in the native or already naturalized European flora, which stem from genera for which hybridization is documented) and which are represented by > 50 occurrence records in GBIF together with their life form (cf. Tab. 3) and their native range (GloNAF; van Kleunen *et al.*, 2015; https://glonaf.org/).

| **spec** | **life form** | **native range** | **GBIF points** |
| --- | --- | --- | --- |
| *Chenopodium quinoa* | annual | S-America | 96 |
| *Eucalyptus cinerea* | tree | Australia | 84 |
| *Eucalyptus cladocalyx* | tree | Australia | 88 |
| *Eucalyptus cornuta* | tree | Australia | 78 |
| *Eucalyptus dalrympleana* | tree | Australia | 277 |
| *Eucalyptus goniocalyx* | tree | Australia | 296 |
| *Eucalyptus leucoxylon* | tree | Australia | 382 |
| *Eucalyptus nitens* | tree | Australia | 73 |
| *Eucalyptus ovata* | tree | Australia | 407 |
| *Eucalyptus rubida* | tree | Australia | 405 |
| *Euphorbia balsamifera* | shrub | Africa (+ Canary Islands) | 69 |
| *Euphorbia leucocephala* | shrub | N-America, S-America | 52 |
| *Euphorbia mauritanica* | herb | Africa | 175 |
| *Euphorbia tithymaloides* | shrub | N-America, S-America | 188 |
| *Fraxinus nigra* | tree | N-America | 118 |
| *Fraxinus uhdei* | tree | N-America, S-America | 151 |
| *Linaria maroccana* | herb | Africa | 206 |
| *Lonicera sempervirens* | herb | N-America | 123 |
| *Malus prunifolia* | tree | Asia | 59 |
| *Populus acuminata* | tree | N-America | 65 |
| *Prunus munsoniana* | tree | N-America, | 57 |
| *Prunus pumila* | shrub | N-America | 152 |
| *Prunus salicina* | tree | Asia | 90 |
| *Pyrus calleryana* | tree | Asia | 88 |
| *Pyrus pyrifolia* | tree | Asia | 102 |
| *Rhododendron japonicum* | shrub | HYBRID | 58 |
| *Senecio radicans* | herb | Africa | 57 |
| *Senecio tamoides* | herb | Africa | 73 |
| *Solidago ptarmicoides* | herb | N-America | 89 |
| *Tamarix aphylla* | tree | Africa, Asia | 205 |
| *Tamarix chinensis* | tree | Asia | 216 |
| *Ulmus parvifolia* | tree | Asia | 146 |
| *Viola hederacea* | herb | Asia | 354 |
| *Viola sororia* | herb | N-America | 849 |

Table S3: Reclassification of life forms reported in the Global Naturalized Flora database (GloNAF; van Kleunen *et al.*, 2015; https://glonaf.org/) used for assessing correspondence between alien garden plants and potential hybridization partners in the resident flora of Europe.

| **Life form** | **Conversion** |
| --- | --- |
| phanerophyte, macrophanerophyte, | tree |
| hemiphanerophyte, nanophanerophyte, shrub | shrub |
| shrub/vine | shrub/vine |
| Chamaephyte | scrub |
| herb, succulent herb | herb |
| forb/vine, herb + forb/herb, geophyte, forb/herb, forb | forb/herb |
| forb annual, therophyte | annual |

Table S4: Linear Mixed Effects Models (LMMs) testing either climate-driven changes in the overlap of climatically suitable ranges (by 2050-2100) of alien garden plants and their congeners in the naturalized and native flora of Europe; or climate-driven changes in the number of cells suitable either to the garden plants or their congeners. Overlap was measured by the True Skill statistic (TSS) and the number of overlapping cells. The column “model” gives the representation of the model in the statistical programming language R. The other columns document fixed effects estimates (est) with lower and upper .95 confidence intervals (lower; upper), standard error (std.error), degrees of freedom (df), t-values and p-values. Significant models (p-value < 0.05) are in bold.

| Model | lower | est | upper | std.error | df | t-value | p-value |
| --- | --- | --- | --- | --- | --- | --- | --- |
| TSS |  |  |  |  |  |  |  |
| **RCP2.6/BASE ~ 1\| genus** | **-0.043** | **-0.020** | **0.004** | **0.009** | **18** | **-2.488** | **0.023*** |
| **RCP4.5/ BASE ~ 1\| genus** | **-0.049** | **-0.026** | **0.004** | **0.011** | **18** | **-2.465** | **0.024*** |
| **RCP8.5/ BASE ~ 1\| genus** | **-0.096** | **-0.061** | **-0.025** | **0.017** | **18** | **-3.622** | **0.0019*** |
| *Cell overlap* |  |  |  |  |  |  |  |
| RCP2.6/BASE ~ 1\| genus | -183 | -6 | 171 | 83 | 15 | -0.071 | 0.944 |
| RCP4.5/ BASE ~ 1\| genus | -679 | -163 | 354 | 242 | 15 | -0.671 | 0.512 |
| RCP8.5/ BASE ~ 1\| genus | -434 | -319 | 1073 | 354 | 15 | 0.903 | 0.381 |
| *Cells natives* |  |  |  |  |  |  |  |
| **RCP2.6/ BASE ~ 1\| genus** | **264** | **510** | **756** | **125** | **157** | **4.088** | **0.0001*** |
| **RCP4.5/ BASE ~ 1\| genus** | **-1621** | **-1041** | **-460** | **294** | **157** | **-3.540** | **0.0005*** |
| RCP8.5/ BASE ~ 1\| genus | -526 | 71 | 667 | 294 | 157 | -0.234 | 0.815 |
| *Cells potential invasives* |  |  |  |  |  |  |  |
| RCP2.6/ BASE ~ 1\| genus | -226 | 195 | 617 | 200 | 18 | 0.975 | 0.342 |
| RCP4.5/ BASE ~ 1\| genus | -572 | 213 | 998 | 374 | 18 | 0.569 | 0.576 |
| **RCP8.5/ BASE ~ 1\| genus** | **-268** | **1713** | **3429** | **826** | **18** | **2.098** | **0.05*** |

Table S5: Range overlap of 34 alien garden plants and their native and already naturalized European congeners under current climate and three climate change scenarios (by 2050-2100) measured as number of cells potentially suitable to both species in each pair. Numbers represent averages over all possible combinations of each of the listed garden plants with each of their possible congeneric species (see Table S6).

| species | base | RCP2.6 | RCP4.5 | RCP8.5 |
| --- | --- | --- | --- | --- |
| *Chenopodium quinoa* | 4710 | 3912 | 3478 | 3622 |
| *Eucalyptus cinerea* | 731 | 1263 | 1411 | 1638 |
| *Eucalyptus cladocalyx* | 975 | 705 | 344 | 374 |
| *Eucalyptus cornuta* | 219 | 175 | 95 | 0 |
| *Eucalyptus dalrympleana* | 373 | 577 | 631 | 462 |
| *Eucalyptus goniocalyx* | 453 | 914 | 670 | 48 |
| *Eucalyptus leucoxylon* | 651 | 862 | 594 | 630 |
| *Eucalyptus nitens* | 513 | 842 | 874 | 399 |
| *Eucalyptus ovata* | 774 | 1217 | 939 | 236 |
| *Eucalyptus rubida* | 186 | 237 | 122 | 0 |
| *Euphorbia balsamifera* | 0 | 0 | 0 | 0 |
| *Euphorbia leucocephala* | 0 | 0 | 0 | 0 |
| *Euphorbia mauritanica* | 44 | 54 | 9 | 1 |
| *Euphorbia tithymaloides* | 16 | 7 | 6 | 0 |
| *Fraxinus nigra* | 32 | 102 | 86 | 766 |
| *Fraxinus uhdei* | 0 | 0 | 0 | 0 |
| *Linaria maroccana* | 9660 | 10280 | 8104 | 7947 |
| *Lonicera sempervirens* | 977 | 1458 | 1888 | 9 |
| *Malus prunifolia* | 7626 | 3765 | 4498 | 2 |
| *Populus acuminata* | 0 | 0 | 0 | 0 |
| *Prunus munsoniana* | 1 | 60 | 240 | 88 |
| *Prunus pumila* | 0 | 0 | 0 | 0 |
| *Prunus salicina* | 1609 | 1636 | 1951 | 844 |
| *Pyrus calleryana* | 2083 | 1924 | 2775 | 3170 |
| *Pyrus pyrifolia* | 15 | 24 | 24 | 0 |
| *Rhododendron japonicum* | 761 | 919 | 1262 | 1454 |
| *Senecio radicans* | 133 | 81 | 35 | 0 |
| *Senecio tamoides* | 110 | 153 | 170 | 0 |
| *Solidago ptarmicoides* | 216 | 353 | 526 | 1168 |
| *Tamarix aphylla* | 3357 | 2812 | 2758 | 434 |
| *Tamarix chinensis* | 1338 | 1106 | 897 | 690 |
| *Ulmus parvifolia* | 1963 | 2699 | 3703 | 2445 |
| *Viola hederacea* | 283 | 336 | 375 | 334 |
| *Viola sororia* | 1357 | 1106 | 1268 | 649 |

Table S6: List of the two species sets modelled: 1) 34 alien garden plants 2) 173 native or already naturalized plant species of Europe which are congeneric and share life forms with the 34 alien garden plants listed in Table S2, and which are represented in GBIF by > 50 occurrences. Further the table reflects model evaluation statistics for all modelled species. Reported is the mean TSS over all replicates and the percentage of replicates that have a TSS < 0.5, respectively, for each modelling technique.

| **species** | **model** | **GLM** | | **GAM** | | **RF** | | **GBM** | |
| --- | --- | --- | --- | --- | --- | --- | --- | --- | --- |
|  | **life form** | **TSS** | **%< 0.5** | **TSS** | **%< 0.5** | **TSS** | **%< 0.5** | **TSS** | **%< 0.5** |
| *Chenopodium quinoa* | annual | 0.835 | 0.0 | 0.778 | 0.0 | 0.900 | 0.0 | 0.902 | 0.0 |
| *Eucalyptus cinerea* | tree | 0.921 | 0.0 | 0.897 | 0.0 | 0.910 | 0.0 | 0.908 | 0.0 |
| *Eucalyptus cladocalyx* | tree | 0.957 | 0.0 | 0.949 | 0.0 | 0.939 | 0.0 | 0.933 | 0.0 |
| *Eucalyptus cornuta* | tree | 0.975 | 0.0 | 0.955 | 0.0 | 0.979 | 0.0 | 0.971 | 3.3 |
| *Eucalyptus dalrympleana* | tree | 0.992 | 0.0 | 0.987 | 0.0 | 0.987 | 0.0 | 0.982 | 0.0 |
| *Eucalyptus goniocalyx* | tree | 0.977 | 0.0 | 0.986 | 0.0 | 0.978 | 0.0 | 0.975 | 0.0 |
| *Eucalyptus leucoxylon* | tree | 0.961 | 0.0 | 0.968 | 0.0 | 0.979 | 0.0 | 0.976 | 0.0 |
| *Eucalyptus nitens* | tree | 0.875 | 0.0 | 0.879 | 0.0 | 0.949 | 0.0 | 0.942 | 0.0 |
| *Eucalyptus ovata* | tree | 0.979 | 0.0 | 0.983 | 0.0 | 0.981 | 0.0 | 0.979 | 0.0 |
| *Eucalyptus rubida* | tree | 0.974 | 0.0 | 0.978 | 0.0 | 0.978 | 0.0 | 0.971 | 0.0 |
| *Euphorbia balsamifera* | shrub | 0.907 | 0.0 | 0.897 | 0.0 | 0.933 | 0.0 | 0.919 | 0.0 |
| *Euphorbia leucocephala* | shrub | 0.777 | 0.0 | 0.837 | 0.0 | 0.793 | 0.0 | 0.800 | 0.0 |
| *Euphorbia mauritanica* | herb | 0.942 | 0.0 | 0.960 | 0.0 | 0.959 | 0.0 | 0.947 | 0.0 |
| *Euphorbia tithymaloides* | shrub | 0.777 | 0.0 | 0.798 | 0.0 | 0.817 | 0.0 | 0.813 | 0.0 |
| *Fraxinus nigra* | tree | 0.947 | 0.0 | 0.950 | 0.0 | 0.964 | 0.0 | 0.951 | 0.0 |
| *Fraxinus uhdei* | tree | 0.831 | 0.0 | 0.822 | 0.0 | 0.889 | 0.0 | 0.881 | 0.0 |
| *Linaria maroccana* | herb | 0.824 | 0.0 | 0.854 | 0.0 | 0.915 | 0.0 | 0.906 | 0.0 |
| *Lonicera sempervirens* | herb | 0.924 | 0.0 | 0.933 | 0.0 | 0.967 | 0.0 | 0.961 | 0.0 |
| *Malus prunifolia* | tree | 0.909 | 0.0 | 0.766 | 0.0 | 0.892 | 0.0 | 0.850 | 0.0 |
| *Populus acuminata* | tree | 0.972 | 0.0 | 0.970 | 0.0 | 0.959 | 0.0 | 0.951 | 0.0 |
| *Prunus munsoniana* | tree | 0.938 | 0.0 | 0.842 | 0.0 | 0.964 | 0.0 | 0.970 | 0.0 |
| *Prunus pumila* | shrub | 0.910 | 0.0 | 0.936 | 0.0 | 0.936 | 0.0 | 0.926 | 0.0 |
| *Prunus salicina* | tree | 0.781 | 0.0 | 0.767 | 0.0 | 0.898 | 0.0 | 0.880 | 0.0 |
| *Pyrus calleryana* | tree | 0.802 | 0.0 | 0.870 | 0.0 | 0.868 | 0.0 | 0.850 | 0.0 |
| *Pyrus pyrifolia* | tree | 0.870 | 0.0 | 0.790 | 0.0 | 0.888 | 0.0 | 0.872 | 0.0 |
| *Rhododendron japonicum* | shrub | 0.893 | 0.0 | 0.792 | 0.0 | 0.947 | 0.0 | 0.922 | 0.0 |
| *Senecio radicans* | herb | 0.844 | 0.0 | 0.768 | 0.0 | 0.958 | 0.0 | 0.939 | 0.0 |
| *Senecio tamoides* | herb | 0.940 | 0.0 | 0.950 | 0.0 | 0.969 | 0.0 | 0.951 | 0.0 |
| *Solidago ptarmicoides* | herb | 0.861 | 0.0 | 0.845 | 0.0 | 0.867 | 0.0 | 0.859 | 0.0 |
| *Tamarix aphylla* | tree | 0.727 | 0.0 | 0.758 | 0.0 | 0.834 | 0.0 | 0.826 | 0.0 |
| *Tamarix chinensis* | tree | 0.722 | 0.0 | 0.799 | 0.0 | 0.836 | 0.0 | 0.824 | 0.0 |
| *Ulmus parvifolia* | tree | 0.827 | 0.0 | 0.732 | 0.0 | 0.815 | 0.0 | 0.817 | 0.0 |
| *Viola hederacea* | herb | 0.978 | 0.0 | 0.976 | 0.0 | 0.986 | 0.0 | 0.974 | 0.0 |
| *Viola sororia* | herb | 0.860 | 0.0 | 0.863 | 0.0 | 0.925 | 0.0 | 0.893 | 0.0 |
| *Chenopodium album* | annual | 0.881 | 0.0 | 0.907 | 0.0 | 0.935 | 0.0 | 0.909 | 0.0 |
| *Chenopodium capitatum* | annual | 0.724 | 0.0 | 0.794 | 0.0 | 0.887 | 0.0 | 0.848 | 0.0 |
| *Chenopodium ficifolium* | annual | 0.917 | 0.0 | 0.944 | 0.0 | 0.969 | 0.0 | 0.960 | 0.0 |
| *Chenopodium foliosum* | annual | 0.809 | 0.0 | 0.831 | 0.0 | 0.889 | 0.0 | 0.876 | 0.0 |
| *Chenopodium glaucum* | annual | 0.820 | 0.0 | 0.830 | 0.0 | 0.925 | 0.0 | 0.891 | 0.0 |
| *Chenopodium hybridum* | annual | 0.955 | 0.0 | 0.948 | 0.0 | 0.963 | 0.0 | 0.961 | 0.0 |
| *Chenopodium murale* | annual | 0.801 | 0.0 | 0.850 | 0.0 | 0.909 | 0.0 | 0.887 | 0.0 |
| *Chenopodium opulifolium* | annual | 0.867 | 0.0 | 0.884 | 0.0 | 0.932 | 0.0 | 0.898 | 0.0 |
| *Chenopodium polyspermum* | annual | 0.930 | 0.0 | 0.946 | 0.0 | 0.973 | 0.0 | 0.967 | 0.0 |
| *Chenopodium rubrum* | annual | 0.902 | 0.0 | 0.919 | 0.0 | 0.956 | 0.0 | 0.941 | 0.0 |
| *Chenopodium strictum* | annual | 0.796 | 0.0 | 0.842 | 0.0 | 0.925 | 0.0 | 0.878 | 0.0 |
| *Chenopodium suecicum* | annual | 0.942 | 0.0 | 0.956 | 0.0 | 0.972 | 0.0 | 0.964 | 0.0 |
| *Chenopodium urbicum* | annual | 0.914 | 0.0 | 0.914 | 0.0 | 0.956 | 0.0 | 0.929 | 0.0 |
| *Chenopodium vulvaria* | annual | 0.883 | 0.0 | 0.889 | 0.0 | 0.941 | 0.0 | 0.933 | 0.0 |
| *Eucalyptus botryoides* | tree | 0.913 | 0.0 | 0.896 | 0.0 | 0.940 | 0.0 | 0.928 | 0.0 |
| *Eucalyptus camaldulensis* | tree | 0.788 | 0.0 | 0.859 | 0.0 | 0.887 | 0.0 | 0.881 | 0.0 |
| *Eucalyptus globulus* | tree | 0.888 | 0.0 | 0.891 | 0.0 | 0.957 | 0.0 | 0.953 | 0.0 |
| *Eucalyptus gomphocephalus* | tree | 0.885 | 0.0 | 0.883 | 0.0 | 0.959 | 0.0 | 0.957 | 0.0 |
| *Eucalyptus resinifer* | tree | 0.911 | 0.0 | 0.887 | 0.0 | 0.936 | 0.0 | 0.933 | 0.0 |
| *Eucalyptus robusta* | tree | 0.888 | 0.0 | 0.859 | 0.0 | 0.898 | 0.0 | 0.895 | 0.0 |
| *Eucalyptus rudis* | tree | 0.892 | 0.0 | 0.898 | 0.0 | 0.936 | 0.0 | 0.933 | 0.0 |
| *Eucalyptus tereticornis* | tree | 0.924 | 0.0 | 0.938 | 0.0 | 0.946 | 0.0 | 0.937 | 0.0 |
| *Eucalyptus viminalis* | tree | 0.962 | 0.0 | 0.959 | 0.0 | 0.972 | 0.0 | 0.962 | 0.0 |
| *Euphorbia acanthothamnos* | scrub | 0.926 | 0.0 | 0.993 | 0.0 | 0.970 | 0.0 | 0.955 | 0.0 |
| *Euphorbia amygdaloides* | scrub | 0.958 | 0.0 | 0.965 | 0.0 | 0.979 | 0.0 | 0.969 | 0.0 |
| *Euphorbia angulata* | forb/herb | 0.943 | 0.0 | 0.973 | 0.0 | 0.969 | 0.0 | 0.966 | 0.0 |
| *Euphorbia biumbellata* | forb/herb | 0.946 | 0.0 | 0.920 | 0.0 | 0.956 | 0.0 | 0.949 | 0.0 |
| *Euphorbia boetica* | forb/herb | 0.939 | 0.0 | 0.940 | 0.0 | 0.982 | 0.0 | 0.968 | 0.0 |
| *Euphorbia brittingeri* | shrub | 0.970 | 0.0 | 0.978 | 0.0 | 0.967 | 0.0 | 0.963 | 0.0 |
| *Euphorbia characias* | forb/shrub | 0.957 | 0.0 | 0.952 | 0.0 | 0.969 | 0.0 | 0.962 | 0.0 |
| *Euphorbia clementei* | forb/herb | 0.980 | 0.0 | 0.927 | 0.0 | 0.973 | 0.0 | 0.973 | 0.0 |
| *Euphorbia cyparissias* | forb/herb | 0.912 | 0.0 | 0.932 | 0.0 | 0.962 | 0.0 | 0.953 | 0.0 |
| *Euphorbia dendroides* | shrub | 0.955 | 0.0 | 0.932 | 0.0 | 0.963 | 0.0 | 0.955 | 0.0 |
| *Euphorbia dracunculoides* | forb/herb | 0.761 | 0.0 | 0.777 | 0.0 | 0.810 | 0.0 | 0.810 | 0.0 |
| *Euphorbia dulcis* | forb/herb | 0.946 | 0.0 | 0.947 | 0.0 | 0.971 | 0.0 | 0.963 | 0.0 |
| *Euphorbia duvalii* | forb/herb | 0.992 | 0.0 | 0.974 | 0.0 | 0.993 | 0.0 | 0.974 | 0.0 |
| *Euphorbia epithymoides* | forb/herb | 0.913 | 0.0 | 0.909 | 0.0 | 0.949 | 0.0 | 0.940 | 0.0 |
| *Euphorbia esula* | forb/herb | 0.858 | 0.0 | 0.854 | 0.0 | 0.924 | 0.0 | 0.899 | 0.0 |
| *Euphorbia helioscopia* | forb/herb | 0.939 | 0.0 | 0.944 | 0.0 | 0.965 | 0.0 | 0.955 | 0.0 |
| *Euphorbia humifusa* | forb/herb | 0.859 | 0.0 | 0.868 | 0.0 | 0.879 | 0.0 | 0.881 | 0.0 |
| *Euphorbia hyberna* | shrub | 0.962 | 0.0 | 0.957 | 0.0 | 0.979 | 0.0 | 0.971 | 0.0 |
| *Euphorbia isatidifolia* | forb/herb | 0.940 | 0.0 | 0.939 | 0.0 | 0.984 | 0.0 | 0.972 | 0.0 |
| *Euphorbia lathyris* | forb/herb | 0.920 | 0.0 | 0.927 | 0.0 | 0.962 | 0.0 | 0.958 | 0.0 |
| *Euphorbia minuta* | forb/herb | 0.956 | 0.0 | 0.972 | 0.0 | 0.967 | 0.0 | 0.960 | 0.0 |
| *Euphorbia myrsinites* | forb/herb | 0.897 | 0.0 | 0.877 | 0.0 | 0.916 | 0.0 | 0.907 | 0.0 |
| *Euphorbia nevadensis* | forb/herb | 0.984 | 0.0 | 0.967 | 0.0 | 0.991 | 0.0 | 0.987 | 0.0 |
| *Euphorbia nicaeensis* | forb/herb | 0.924 | 0.0 | 0.924 | 0.0 | 0.961 | 0.0 | 0.955 | 0.0 |
| *Euphorbia oblongata* | forb/herb | 0.951 | 0.0 | 0.911 | 0.0 | 0.925 | 0.0 | 0.914 | 0.0 |
| *Euphorbia palustris* | forb/herb | 0.940 | 0.0 | 0.947 | 0.0 | 0.961 | 0.0 | 0.951 | 0.0 |
| *Euphorbia paralias* | forb/herb | 0.964 | 0.0 | 0.966 | 0.0 | 0.956 | 0.0 | 0.944 | 0.0 |
| *Euphorbia peplis* | forb/herb | 0.950 | 0.0 | 0.952 | 0.0 | 0.941 | 0.0 | 0.933 | 0.0 |
| *Euphorbia pithyusa* | scrub | 0.930 | 0.0 | 0.919 | 0.0 | 0.974 | 0.0 | 0.968 | 0.0 |
| *Euphorbia polygalifolia* | forb/herb | 0.836 | 0.0 | 0.893 | 0.0 | 0.905 | 0.0 | 0.885 | 0.0 |
| *Euphorbia portlandica* | shrub | 0.983 | 0.0 | 0.979 | 0.0 | 0.976 | 0.0 | 0.971 | 0.0 |
| *Euphorbia prostrata* | forb/herb | 0.594 | 0.0 | 0.692 | 0.0 | 0.812 | 0.0 | 0.774 | 0.0 |
| *Euphorbia pubescens* | forb/herb | 0.874 | 0.0 | 0.924 | 0.0 | 0.908 | 0.0 | 0.897 | 0.0 |
| *Euphorbia serrata* | forb/herb | 0.905 | 0.0 | 0.961 | 0.0 | 0.967 | 0.0 | 0.960 | 0.0 |
| *Euphorbia serrulata* | forb/herb | 0.781 | 0.0 | 0.833 | 0.0 | 0.901 | 0.0 | 0.888 | 0.0 |
| *Euphorbia spinosa* | scrub | 0.968 | 0.0 | 0.940 | 0.0 | 0.970 | 0.0 | 0.961 | 0.0 |
| *Euphorbia squamigera* | shrub | 0.981 | 0.0 | 0.975 | 0.0 | 0.968 | 0.0 | 0.961 | 0.0 |
| *Euphorbia taurinensis* | forb/herb | 0.860 | 0.0 | 0.854 | 0.0 | 0.902 | 0.0 | 0.897 | 0.0 |
| *Euphorbia terracina* | forb/herb | 0.952 | 0.0 | 0.955 | 0.0 | 0.966 | 0.0 | 0.956 | 0.0 |
| *Fraxinus angustifolia* | tree | 0.959 | 0.0 | 0.960 | 0.0 | 0.966 | 0.0 | 0.959 | 0.0 |
| *Fraxinus excelsior* | tree | 0.957 | 0.0 | 0.960 | 0.0 | 0.977 | 0.0 | 0.969 | 0.0 |
| *Fraxinus ornus* | tree | 0.934 | 0.0 | 0.944 | 0.0 | 0.962 | 0.0 | 0.957 | 0.0 |
| *Fraxinus pennsylvanica* | tree | 0.807 | 0.0 | 0.835 | 0.0 | 0.927 | 0.0 | 0.900 | 0.0 |
| *Linaria aeruginea* | forb/herb | 0.953 | 0.0 | 0.961 | 0.0 | 0.965 | 0.0 | 0.958 | 0.0 |
| *Linaria alpina* | forb/herb | 0.940 | 0.0 | 0.935 | 0.0 | 0.971 | 0.0 | 0.968 | 0.0 |
| *Linaria angustissima* | forb/herb | 0.987 | 0.0 | 0.993 | 0.0 | 0.972 | 0.0 | 0.960 | 0.0 |
| *Linaria canadensis* | annual | 0.781 | 0.0 | 0.778 | 0.0 | 0.880 | 0.0 | 0.864 | 0.0 |
| *Linaria genistifolia* | forb/herb | 0.931 | 0.0 | 0.925 | 0.0 | 0.906 | 0.0 | 0.888 | 0.0 |
| *Linaria incarnata* | forb/herb | 0.860 | 0.0 | 0.875 | 0.0 | 0.880 | 0.0 | 0.886 | 0.0 |
| *Linaria purpurea* | forb/herb | 0.971 | 0.0 | 0.975 | 0.0 | 0.974 | 0.0 | 0.967 | 0.0 |
| *Linaria repens* | forb/herb | 0.941 | 0.0 | 0.965 | 0.0 | 0.975 | 0.0 | 0.964 | 0.0 |
| *Linaria saxatilis* | forb/herb | 0.959 | 0.0 | 0.987 | 0.0 | 0.976 | 0.0 | 0.969 | 0.0 |
| *Linaria supina* | forb/herb | 0.911 | 0.0 | 0.924 | 0.0 | 0.964 | 0.0 | 0.947 | 0.0 |
| *Linaria vulgaris* | forb/herb | 0.880 | 0.0 | 0.894 | 0.0 | 0.948 | 0.0 | 0.929 | 0.0 |
| *Lonicera etrusca* | shrub/vine | 0.949 | 0.0 | 0.955 | 0.0 | 0.965 | 0.0 | 0.957 | 0.0 |
| *Lonicera implexa* | shrub/vine | 0.973 | 0.0 | 0.977 | 0.0 | 0.981 | 0.0 | 0.968 | 0.0 |
| *Lonicera japonica* | shrub/vine | 0.833 | 0.0 | 0.860 | 0.0 | 0.934 | 0.0 | 0.915 | 0.0 |
| *Malus domestica* | tree | 0.900 | 0.0 | 0.911 | 0.0 | 0.941 | 0.0 | 0.926 | 0.0 |
| *Malus sylvestris* | tree | 0.931 | 0.0 | 0.953 | 0.0 | 0.974 | 0.0 | 0.962 | 0.0 |
| *Populus alba* | tree | 0.922 | 0.0 | 0.917 | 0.0 | 0.950 | 0.0 | 0.938 | 46.7 |
| *Populus candicans* | tree | 0.984 | 0.0 | 0.992 | 0.0 | 0.977 | 0.0 | 0.961 | 0.0 |
| *Populus canescens* | tree | 0.923 | 0.0 | 0.935 | 0.0 | 0.970 | 0.0 | 0.960 | 0.0 |
| *Populus deltoides* | tree | 0.769 | 0.0 | 0.830 | 0.0 | 0.935 | 0.0 | 0.910 | 0.0 |
| *Populus euphratica* | tree | 0.711 | 0.0 | 0.747 | 0.0 | 0.806 | 0.0 | 0.792 | 0.0 |
| *Populus grandidentata* | tree | 0.960 | 0.0 | 0.942 | 0.0 | 0.968 | 0.0 | 0.960 | 0.0 |
| *Populus nigra* | tree | 0.916 | 0.0 | 0.930 | 0.0 | 0.963 | 0.0 | 0.958 | 0.0 |
| *Populus simonii* | tree | 0.785 | 0.0 | 0.789 | 0.0 | 0.839 | 0.0 | 0.814 | 0.0 |
| *Populus tremula* | tree | 0.922 | 0.0 | 0.943 | 0.0 | 0.964 | 0.0 | 0.956 | 76.7 |
| *Prunus armeniaca* | tree | 0.735 | 0.0 | 0.728 | 0.0 | 0.823 | 0.0 | 0.807 | 0.0 |
| *Prunus avium* | tree | 0.953 | 0.0 | 0.953 | 0.0 | 0.966 | 0.0 | 0.957 | 0.0 |
| *Prunus cerasifera* | tree | 0.912 | 0.0 | 0.924 | 0.0 | 0.967 | 0.0 | 0.964 | 0.0 |
| *Prunus cerasus* | tree | 0.903 | 0.0 | 0.936 | 0.0 | 0.964 | 0.0 | 0.949 | 0.0 |
| *Prunus domestica* | tree | 0.918 | 0.0 | 0.936 | 0.0 | 0.961 | 0.0 | 0.947 | 0.0 |
| *Prunus dulcis* | tree | 0.896 | 0.0 | 0.901 | 0.0 | 0.929 | 0.0 | 0.920 | 0.0 |
| *Prunus fruticosa* | tree | 0.774 | 0.0 | 0.794 | 0.0 | 0.927 | 0.0 | 0.917 | 0.0 |
| *Prunus laurocerasus* | shrub | 0.962 | 0.0 | 0.957 | 0.0 | 0.980 | 0.0 | 0.971 | 0.0 |
| *Prunus lusitanica* | shrub | 0.940 | 0.0 | 0.956 | 0.0 | 0.976 | 0.0 | 0.972 | 0.0 |
| *Prunus mahaleb* | tree | 0.915 | 0.0 | 0.912 | 0.0 | 0.950 | 0.0 | 0.940 | 0.0 |
| *Prunus padus* | tree | 0.927 | 0.0 | 0.932 | 0.0 | 0.969 | 0.0 | 0.955 | 0.0 |
| *Prunus persica* | tree | 0.789 | 0.0 | 0.801 | 0.0 | 0.881 | 0.0 | 0.866 | 0.0 |
| *Prunus prostrata* | shrub | 0.935 | 0.0 | 0.936 | 0.0 | 0.950 | 0.0 | 0.948 | 0.0 |
| *Prunus serotina* | tree | 0.902 | 0.0 | 0.930 | 0.0 | 0.959 | 0.0 | 0.952 | 0.0 |
| *Prunus spinosa* | tree | 0.961 | 0.0 | 0.961 | 0.0 | 0.980 | 0.0 | 0.970 | 0.0 |
| *Prunus virginiana* | tree | 0.801 | 0.0 | 0.825 | 0.0 | 0.928 | 0.0 | 0.874 | 0.0 |
| *Pyrus bourgaeana* | tree | 0.979 | 0.0 | 0.978 | 0.0 | 0.982 | 0.0 | 0.972 | 0.0 |
| *Pyrus pyraster* | tree | 0.967 | 0.0 | 0.966 | 0.0 | 0.980 | 0.0 | 0.972 | 0.0 |
| *Rhododendron ferrugineum* | shrub | 0.978 | 0.0 | 0.964 | 0.0 | 0.983 | 0.0 | 0.972 | 0.0 |
| *Rhododendron hirsutum* | shrub | 0.973 | 0.0 | 0.976 | 0.0 | 0.964 | 0.0 | 0.958 | 0.0 |
| *Rhododendron lapponicum* | shrub | 0.754 | 0.0 | 0.764 | 0.0 | 0.887 | 0.0 | 0.870 | 0.0 |
| *Rhododendron luteum* | shrub | 0.947 | 0.0 | 0.928 | 0.0 | 0.949 | 0.0 | 0.937 | 0.0 |
| *Rhododendron ponticum* | shrub | 0.956 | 0.0 | 0.957 | 0.0 | 0.976 | 0.0 | 0.965 | 0.0 |
| *Senecio cacaliaster* | forb/herb | 0.993 | 0.0 | 0.996 | 0.0 | 0.974 | 0.0 | 0.965 | 0.0 |
| *Senecio carpetanus* | forb/herb | 0.991 | 0.0 | 0.993 | 0.0 | 0.990 | 0.0 | 0.979 | 0.0 |
| *Senecio doronicum* | forb/herb | 0.941 | 0.0 | 0.949 | 0.0 | 0.954 | 0.0 | 0.945 | 0.0 |
| *Senecio erucifolius* | forb/herb | 0.967 | 0.0 | 0.962 | 0.0 | 0.987 | 0.0 | 0.982 | 0.0 |
| *Senecio inaequidens* | forb/herb | 0.969 | 0.0 | 0.971 | 0.0 | 0.980 | 0.0 | 0.973 | 0.0 |
| *Senecio lagascanus* | forb/herb | 0.966 | 0.0 | 0.942 | 0.0 | 0.977 | 0.0 | 0.972 | 0.0 |
| *Senecio lividus* | annual | 0.942 | 0.0 | 0.953 | 0.0 | 0.965 | 0.0 | 0.956 | 0.0 |
| *Senecio nebrodensis* | forb/herb | 0.927 | 0.0 | 0.924 | 0.0 | 0.929 | 0.0 | 0.911 | 0.0 |
| *Senecio nemorensis* | forb/herb | 0.899 | 0.0 | 0.907 | 0.0 | 0.932 | 0.0 | 0.925 | 0.0 |
| *Senecio nevadensis* | forb/herb | 0.954 | 0.0 | 0.985 | 0.0 | 0.968 | 0.0 | 0.955 | 0.0 |
| *Senecio pyrenaicus* | forb/herb | 0.985 | 0.0 | 0.982 | 0.0 | 0.970 | 0.0 | 0.959 | 0.0 |
| *Senecio smithii* | forb/herb | 0.991 | 0.0 | 0.945 | 0.0 | 0.988 | 0.0 | 0.995 | 0.0 |
| *Senecio squalidus* | forb/herb | 0.939 | 0.0 | 0.946 | 0.0 | 0.956 | 0.0 | 0.948 | 0.0 |
| *Senecio sylvaticus* | forb/herb | 0.937 | 0.0 | 0.952 | 0.0 | 0.960 | 0.0 | 0.948 | 0.0 |
| *Senecio viscosus* | forb/herb | 0.934 | 0.0 | 0.956 | 0.0 | 0.970 | 0.0 | 0.964 | 0.0 |
| *Senecio vulgaris* | forb/herb | 0.894 | 0.0 | 0.928 | 0.0 | 0.947 | 0.0 | 0.930 | 0.0 |
| *Solidago canadensis* | forb/herb | 0.898 | 0.0 | 0.905 | 0.0 | 0.946 | 0.0 | 0.935 | 0.0 |
| *Solidago gigantea* | forb/herb | 0.903 | 0.0 | 0.906 | 0.0 | 0.950 | 0.0 | 0.944 | 0.0 |
| *Solidago sempervirens* | forb/herb | 0.885 | 0.0 | 0.895 | 0.0 | 0.912 | 0.0 | 0.905 | 0.0 |
| *Solidago virgaurea* | forb/herb | 0.924 | 0.0 | 0.944 | 0.0 | 0.964 | 0.0 | 0.951 | 0.0 |
| *Tamarix africana* | tree | 0.942 | 0.0 | 0.941 | 0.0 | 0.958 | 0.0 | 0.950 | 0.0 |
| *Tamarix boveana* | tree | 0.991 | 0.0 | 0.992 | 0.0 | 0.990 | 0.0 | 0.990 | 0.0 |
| *Tamarix canariensis* | tree | 0.947 | 0.0 | 0.957 | 0.0 | 0.952 | 0.0 | 0.935 | 0.0 |
| *Tamarix gallica* | tree | 0.898 | 0.0 | 0.901 | 0.0 | 0.916 | 0.0 | 0.905 | 0.0 |
| *Tamarix parviflora* | tree | 0.781 | 0.0 | 0.782 | 0.0 | 0.910 | 0.0 | 0.890 | 0.0 |
| *Tamarix ramosissima* | tree | 0.740 | 0.0 | 0.769 | 0.0 | 0.883 | 0.0 | 0.857 | 0.0 |
| *Ulmus glabra* | tree | 0.965 | 0.0 | 0.968 | 0.0 | 0.967 | 0.0 | 0.961 | 0.0 |
| *Ulmus laevis* | tree | 0.922 | 0.0 | 0.928 | 0.0 | 0.968 | 0.0 | 0.960 | 0.0 |
| *Ulmus minor* | tree | 0.951 | 0.0 | 0.950 | 0.0 | 0.976 | 0.0 | 0.968 | 0.0 |
| *Viola arvensis* | forb/herb | 0.923 | 0.0 | 0.924 | 0.0 | 0.957 | 0.0 | 0.947 | 0.0 |
| *Viola biflora* | forb/herb | 0.927 | 0.0 | 0.940 | 0.0 | 0.960 | 0.0 | 0.950 | 0.0 |
| *Viola bubanii* | forb/herb | 0.992 | 0.0 | 0.993 | 0.0 | 0.969 | 0.0 | 0.960 | 0.0 |
| *Viola calcarata* | forb/herb | 0.956 | 0.0 | 0.952 | 0.0 | 0.946 | 0.0 | 0.934 | 0.0 |
| *Viola canina* | forb/herb | 0.907 | 0.0 | 0.931 | 0.0 | 0.964 | 0.0 | 0.950 | 0.0 |
| *Viola cenisia* | forb/herb | 0.996 | 0.0 | 0.971 | 0.0 | 0.961 | 0.0 | 0.953 | 0.0 |
| *Viola collina* | forb/herb | 0.917 | 0.0 | 0.923 | 0.0 | 0.942 | 0.0 | 0.938 | 0.0 |
| *Viola cornuta* | forb/herb | 0.923 | 0.0 | 0.933 | 0.0 | 0.947 | 0.0 | 0.943 | 0.0 |
| *Viola elatior* | forb/herb | 0.906 | 0.0 | 0.930 | 0.0 | 0.972 | 0.0 | 0.964 | 0.0 |
| *Viola epipsila* | forb/herb | 0.892 | 0.0 | 0.958 | 0.0 | 0.973 | 0.0 | 0.962 | 0.0 |
| *Viola hirta* | forb/herb | 0.976 | 0.0 | 0.980 | 0.0 | 0.987 | 0.0 | 0.981 | 0.0 |
| *Viola jordanii* | forb/herb | 0.864 | 0.0 | 0.832 | 0.0 | 0.957 | 0.0 | 0.953 | 0.0 |
| *Viola kitaibeliana* | forb/herb | 0.939 | 0.0 | 0.947 | 0.0 | 0.964 | 0.0 | 0.950 | 0.0 |
| *Viola lactea* | forb/herb | 0.960 | 0.0 | 0.964 | 0.0 | 0.983 | 0.0 | 0.974 | 0.0 |
| *Viola lutea* | forb/herb | 0.993 | 0.0 | 0.993 | 0.0 | 0.990 | 0.0 | 0.984 | 0.0 |
| *Viola mirabilis* | forb/herb | 0.935 | 0.0 | 0.956 | 0.0 | 0.970 | 0.0 | 0.959 | 0.0 |
| *Viola odorata* | forb/herb | 0.932 | 0.0 | 0.940 | 0.0 | 0.962 | 0.0 | 0.948 | 0.0 |
| *Viola palustris* | forb/herb | 0.907 | 0.0 | 0.937 | 0.0 | 0.966 | 0.0 | 0.952 | 0.0 |
| *Viola parvula* | forb/herb | 0.974 | 0.0 | 0.947 | 0.0 | 0.937 | 0.0 | 0.928 | 0.0 |
| *Viola persicifolia* | forb/herb | 0.960 | 0.0 | 0.977 | 0.0 | 0.980 | 0.0 | 0.973 | 0.0 |
| *Viola pumila* | forb/herb | 0.939 | 0.0 | 0.959 | 0.0 | 0.987 | 0.0 | 0.979 | 0.0 |
| *Viola pyrenaica* | forb/herb | 0.978 | 0.0 | 0.981 | 0.0 | 0.971 | 0.0 | 0.965 | 0.0 |
| *Viola reichenbachiana* | forb/herb | 0.979 | 0.0 | 0.981 | 0.0 | 0.984 | 0.0 | 0.978 | 36.7 |
| *Viola riviniana* | forb/herb | 0.933 | 0.0 | 0.951 | 0.0 | 0.961 | 0.0 | 0.955 | 36.7 |
| *Viola rupestris* | forb/herb | 0.889 | 0.0 | 0.908 | 0.0 | 0.943 | 0.0 | 0.923 | 0.0 |
| *Viola selkirkii* | forb/herb | 0.862 | 0.0 | 0.871 | 0.0 | 0.947 | 0.0 | 0.935 | 0.0 |
| *Viola suavis* | forb/herb | 0.930 | 0.0 | 0.944 | 0.0 | 0.964 | 0.0 | 0.959 | 0.0 |
| *Viola tricolor* | forb/herb | 0.923 | 0.0 | 0.941 | 0.0 | 0.954 | 0.0 | 0.938 | 0.0 |
| *Viola uliginosa* | forb/herb | 0.949 | 0.0 | 0.956 | 0.0 | 0.978 | 0.0 | 0.963 | 0.0 |
| *Viola willkommii* | forb/herb | 0.984 | 0.0 | 0.977 | 0.0 | 0.974 | 0.0 | 0.964 | 0.0 |

**Figure S1**


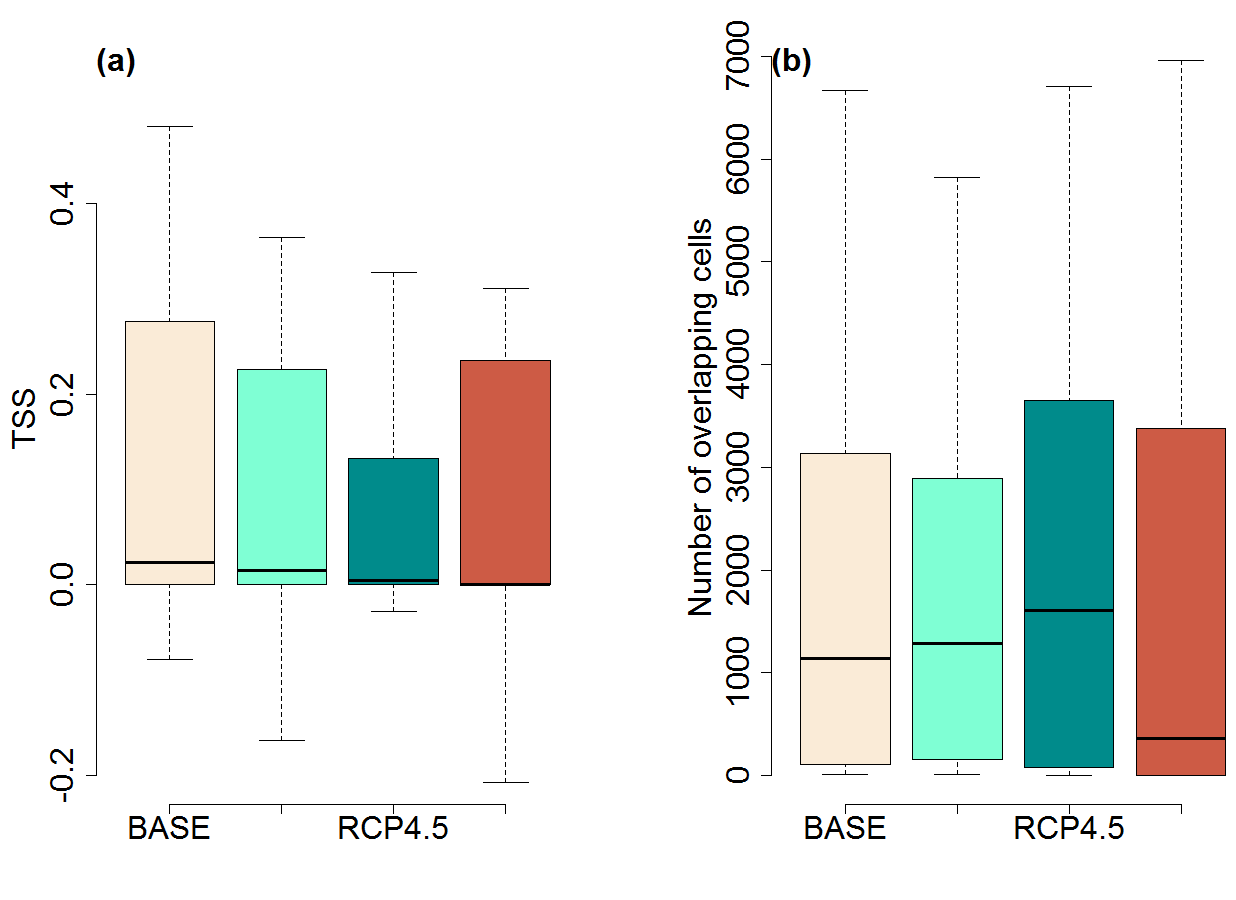


Fig. S1: Mean overlap in areas climatically suitable to 15 alien garden plants and their congeners in the native and naturalized flora of Europe. Only garden plant species native to Africa or Asia and their congeners are included. Overlap was quantified by using True Skill Statistic - TSS (A), or the number of overlapping cells (B), and calculated for current climate (BASE) and under three scenarios of climate change (RCP2.6, RCP4.5, RCP8.5) for the second half of the 21st century (2050-2100).

**Figure S2**


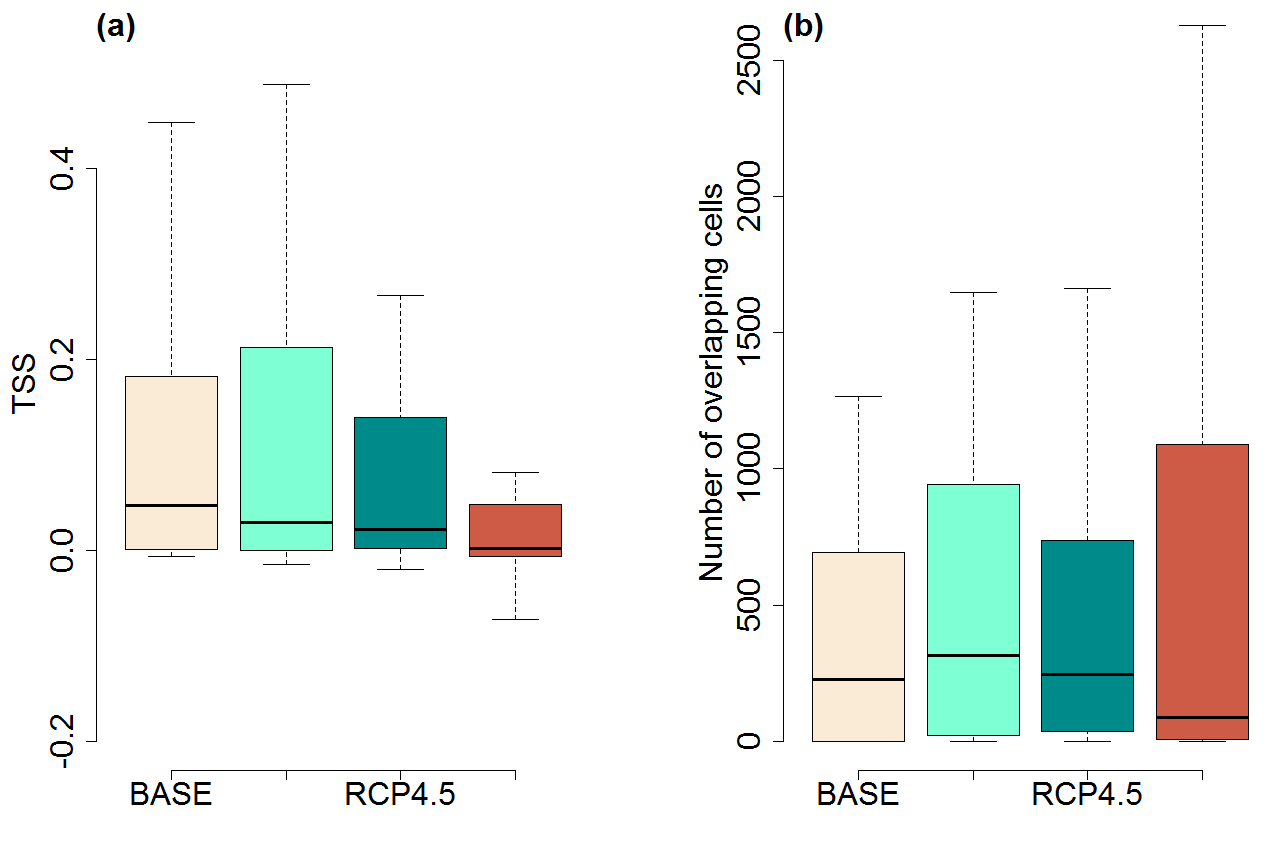


Fig. S2: Mean overlap in areas climatically suitable to 19 alien garden plants and their congeners in the native and naturalized flora of Europe. Only garden plant species not native to Africa or Asia and their congeners are included. Overlap was quantified by using True Skill Statistic - TSS (A), or the number of overlapping cells (B), and calculated for current climate (BASE) and under three scenarios of climate change (RCP2.6, RCP4.5, RCP8.5) for the second half of the 21st century (2050-2100).

**Figure S3**


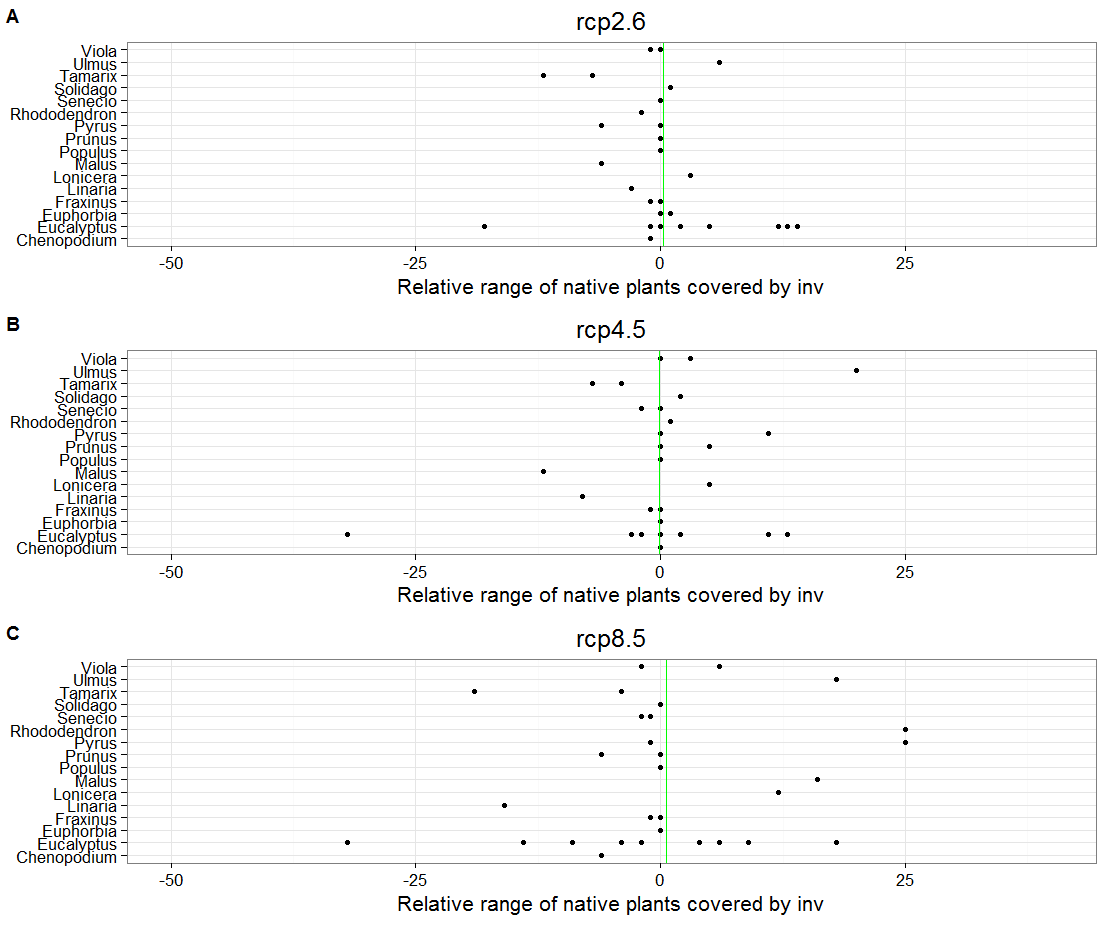


Fig. S3: Climate-driven changes in the share of the ranges of native and naturalized plant species in Europe which is also suitable to congeners among the modelled 34 alien garden plants. Share of range is measured by the ratio of 10 x 10’ cells suitable to both species in a possible species pair. Each point represents the average share of range between one of the 34 garden plants and all its congeners under the respective climate scenario (some points represent more than one pair because of identical values). Values < 0 represent decreasing, values > 0 increasing, values = 0 no change in share The three panels refer to climate change scenarios RCP2.6 (A), RCP4.5 (B) and RCP8.5 (C). The green line represents the mean over all pairs.

Reference:

Barbour, R. C., Potts, B. M., & Vaillancourt, R. E. (2006). Gene flow between introduced and native *Eucalyptus* species: Early-age selection limits invasive capacity of exotic E-ovata x nitens F-1 hybrids. *Forest Ecology* *and Management*, *228*, 206–214.

Barbour, R. C., Potts, B. M., & Vaillancourt, R. E. (2007). Gene flow between introduced and native *Eucalyptus* species: Morphological analysis of Tri-species and backcross hybrids involving E-nitens.*Silvae Genetica*,*56*, 127–133.

Barbour, R. C., Wise, S. L., McKinnon, G. E., Vaillancourt, R. E., Williamson, G. J., & Potts, B. M. (2010). The potential for gene flow from exotic eucalypt plantations into Australia’s rare native eucalypts. *Forest Ecology* *and Management*, *260*, 2079–2087.

Bell, R. L., & Itai, A. (2011). *Pyrus*. In C. Kole (Ed.), *Wild crop relatives: Genomic and breeding resources: Temperate fruits* (pp. 147–177). Berlin,Heidelberg: Springer Berlin Heidelberg.

Lindgren, C., Pearce, C., & Allison, K. (2010). The biology of invasive alien plants in Canada. 11. *Tamarix ramosissima Ledeb.*, *T. chinensis Lour.* and hybrids. *Canadian Journal of Plant Science*, *90*, 111–124.

Pelser, P. B., Abbott, R. J., Comes, H. P., Milton, J. J., Moller, M., Looseley, M. E., … Kadereit, J. W. (2012). The genetic ghost of an invasion past: Colonization and extinction revealed by historical hybridization in *Senecio*. *Molecular Ecology*, *21*, 369–387.

Thomasset, M., Hodkinson, T. R., Restoux, G., Frascaria-Lacoste, N., Douglas, G. C., & Fernandez-Manjarres, J. F. (2014). Thank you for not flowering: Conservation genetics and gene flow analysis of native and non-native populations of Fraxinus (Oleaceae) in Ireland. *Heredity*, *112*,

596–606.

Ward, S. M., Fleischmann, C. E., Turner, M. F., & Sing, S. E. (2009). Hybridization between invasive populations of Dalmatian Toadflax (*Linaria dalmatica*) and Yellow Toadflax (*Linaria vulgaris*). *Invasive Plant* *Science and Management*, *2*, 369–378.

Yamamoto, T., Kimura, T., Shoda, M., Ban, Y., Hayashi, T., & Matsuta, N. (2002). Development of microsatellite markers in the Japanese pear (*Pyrus pyrifolia* Nakai). *Molecular Ecology Notes*, *2*,

14–16.

Zalapa, J. E., Brunet, J., & Guries, R. P. (2010). The extent of hybridization and its impact on the genetic diversity and population structure of an invasive tree, *Ulmus pumila* (Ulmaceae). *Evolutionary Applications*, *3*, 157–168.
